# Significant expansion of the donor pool achieved by utilizing islets of variable quality in the production of allogeneic “Neo-Islets”, 3-D organoids of Mesenchymal Stromal and islet cells, a novel immune-isolating biotherapy for Type I Diabetes

**DOI:** 10.1371/journal.pone.0290460

**Published:** 2023-08-24

**Authors:** Anna M. Gooch, Sabiha S. Chowdhury, Ping M. Zhang, Zhuma M. Hu, Christof Westenfelder

**Affiliations:** 1 SymbioCellTech, LLC, Salt Lake City, Utah, United States of Ameirca; 2 University of Utah, Health Sciences Center, Salt Lake City, Utah, United States of America; Universita degli Studi di Torino, ITALY

## Abstract

Novel biotherapies for Type 1 Diabetes that provide a significantly expanded donor pool and that deliver all islet hormones without requiring anti-rejection drugs are urgently needed. Scoring systems have improved islet allotransplantation outcomes, but their use may potentially result in the waste of valuable cells for novel therapies. To address these issues, we created “Neo-Islets” (NIs), islet-sized organoids, by co-culturing in ultralow adhesion flasks culture-expanded islet (ICs) and Mesenchymal Stromal Cells (MSCs) (x 24 hrs, 1:1 ratio). The MSCs exert powerful immune- and cyto-protective, anti-inflammatory, proangiogenic, and other beneficial actions in NIs. The robust *in vitro* expansion of all islet hormone-producing cells is coupled to their expected progressive de-differentiation mediated by serum-induced cell cycle entry and Epithelial-Mesenchymal Transition (EMT). Re-differentiation *in vivo* of the ICs and resumption of their physiological functions occurs by reversal of EMT and serum withdrawal-induced exit from the cell cycle. Accordingly, we reported that allogeneic, i.p.-administered NIs engraft in the omentum, increase Treg numbers and reestablish permanent normoglycemia in autoimmune diabetic NOD mice without immunosuppression. Our FDA-guided pilot study (INAD 012–0776) in insulin-dependent pet dogs showed similar responses, and both human- and canine-NIs established normoglycemia in STZ-diabetic NOD/SCID mice even though the utilized islets would be scored as unsuitable for transplantation. The present study further demonstrates that islet gene expression profiles (α, β, γ, δ) in human “non-clinical grade” islets obtained from diverse, non-diabetic human and canine donors (n = 6 each) closely correlate with population doublings, and the *in vivo* re-differentiation of endocrine islet cells clearly corresponds with the reestablishment of euglycemia in diabetic mice. Conclusion: human-NIs created from diverse, “non-clinical grade” donors have the potential to greatly expand patient access to this curative therapy of T1DM, facilitated by the efficient *in vitro* expansion of ICs that can produce ~ 270 therapeutic NI doses per donor for 70 kg recipients.

## Introduction

A globally available therapeutic that re-establishes insulin independence in patients with Type 1 Diabetes (T1DM) while also not requiring potentially toxic antirejection drugs is urgently needed in order to improve the quality of life and prognosis of millions of insulin-dependent diabetic patients worldwide. Effective current treatments are allogeneic pancreas or islet transplants, both of which can provide endogenous insulin replacement and normalized glycemic control. However, the limited availability of donors, the need for repeated islet transplants, and the reliance on often toxic antirejection drugs limits the safety and use of such therapies [1, 2]. Because both allogeneic islet and pancreas transplants, and several experimental, allogeneic, insulin producing, stem- or precursor cell-based therapies still depend on the life-long use of potentially harmful antirejection drugs [3–6], auto- and allo-immune isolation of beta-like cells is being tested with various encapsulation technologies. Unfortunately, some of these are still prone to foreign body reactions, amyloid accumulation, release of alloantigens and consequently have frequently failed [7–11]. To address these issues, we created “Neo-Islets” (NIs), islet-sized organoids, by co-aggregating culture-expanded islet cells (ICs) and Mesenchymal Stromal Cells (MSCs) in a 1:1 ratio. The MSCs exert in the microenvironment of the engrafted NIs powerful immune- and cyto-protective, anti-inflammatory, proangiogenic, and other beneficial actions.

Accordingly, we previously demonstrated that allogeneic NIs, when administered intraperitoneally (i.p.), restore permanent euglycemia in diabetic NOD mice after spontaneous omental engraftment, and up-regulation of Tregs. Importantly, this is accomplished without the use of encapsulation devices or antirejection drugs [12]. This therapy establishes insulin independence through redifferentiation of the culture expansion-induced partially dedifferentiated IC component of the NIs, which includes α, β, γ, and δ cells, the process of which is diagrammed in the **S1 Fig**. Of further clinical importance is the fact that retrieval of engrafted NIs from euglycemic NOD mice, when potentially required, results in the prompt return of the pre-treatment diabetic state. In addition, NI administration to both non-diabetic and diabetic mice and dogs has never resulted in hypoglycemia, a response that clearly demonstrates the physiological metabolic control that NIs provide [12]. Comparable results to those in mice were obtained when canine NIs (cNIs), composed of Adipose-derived Stromal Cells (ASCs) and culture expanded canine islet cells were administered i.p. to Streptozotocin (STZ)-diabetic NOD/SCID mice. Such mice were rendered euglycemic long term through exclusive secretion of canine insulin (ELISA) while subsequent retrieval of omentally engrafted cNIs resulted in prompt return of hyperglycemia [12]. Similar results were obtained using human derived NIs in STZ-diabetic NOD/SCID mice, where euglycemia was achieved through secretion of human insulin (ELISA) [13], indicating that NI therapy is clearly translatable to multiple species. Indeed, in order to further test this promising biotherapeutic in a larger animal model and a more real-world setting, we are conducting an FDA-guided pilot study (INAD 012–776), treating spontaneously insulin-dependent pet dogs with allogeneic canine NIs (cNIs), using adapted protocols that were shown to be effective in NOD mice. Demonstrating the translatable nature of this therapy, cNI-treated diabetic dogs that received the initial cNI dose have shown a significant and durable improvement in glycemic control, coupled with a significant reduction in the need for insulin, both achieved without the use of encapsulation devices or antirejection drugs [14]. So far, such effects have lasted at least 3 years, and this without serious adverse events or allo-immune responses to NIs. There was no karyotypic instability of culture-expanded MSCs/ASCs and islet cells, and there was no evidence of *in vivo* oncogenic transformation or proliferation of cells that are delivered by NIs. And significantly, this therapy maintained normal renal and liver functions, complete blood counts, body weights and blood pressures in all study dogs (Loy Son, et al., [Unpublished]).

The engineering of NIs through co-aggregation of culture expanded, partially de-differentiated ICs and cultured MSCs/ASCs in a 1:1 ratio differs from approaches other investigators have taken. Specifically, the well known beneficial islet transplant outcomes that were achieved by co-administered MSCs, either as a combination or by covering the islets with MSCs, were clearly demonstrated by several groups [15–21]. The normal islet contains 2–3% MSCs that gradually lose, due to glucotoxicity, their locally expressed cytoprotective and anti-inflammatory potencies in the diabetic state [22]. In order to significantly boost in the microenvironment of the islets the complex protective actions of MSCs, we increased the percentage of healthy MSCs that are stochastically distributed throughout the NIs to ~50%. This achieved the desired protective effects of allogeneic NIs in the durable therapy of T1DM in rodent models and dogs [12–14], and this without auto- and allogeneic immune responses, thereby durably eliminating the need for potentially harmful anti-rejection drugs. Furthermore, NIs contain by design all major endocrine cells of islets, which provides better metabolic control compared to what is achieved with biotherapies that deliver only insulin [23]. Furthermore, the islet hormone delivery pathway from omentally engrafted NIs mirrors that from the pancreas, i.e., physiological transport into the portal system of the liver and not via subcutaneous or intramuscular route. The latter can both induce insulin resistance and vascular insulin toxicity [24–26].

The positive results in our three, proof-of-concept studies conducted in two species strongly indicate that this therapy could be further translated to the treatment of human mellitus T1DM. However, like islet and pancreas transplants, NI therapy relies on islet donors, albeit to a much smaller extent due to the use of culture expanded islet cells rather than the islets themselves.

The Neo-Islet (NI) technology utilizes the culture expansion of islets, which results in the outgrowth and robust *in vitro* expansion of the major pancreatic islet hormone-producing cells (α, β, γ, δ), a response that is coupled to their expected progressive de-differentiation. This well documented *in vitro* response is simultaneously carried out by serum-induced cell cycle entry and cell proliferation, reversible Epithelial-Mesenchymal Transition (EMT), and by the triggering of the reversible Regenerative Repair Program in sublethally, usually ischemically injured islet cells during islet isolation. Re-differentiation of the cultured islet cells both *in vitro* and *in vivo* occurs by reversal of EMT and exit from the cell cycle upon withdrawal of serum-containing culture media, and completion of the Regenerative Repair Program [27–31]. Of note is the fact that the MSCs in the NIs besides blocking apoptosis, inflammation, fibrosis, adverse actions of Reactive Oxygen Species and auto- and allo-immune attacks on allogeneic cells in the NIs, have the capacity to also support the redifferentiation of the ICs in the NIs, mediated by pro-angiogenic factors and miR-375 release and others mechanisms [19–21, 32–35].

The further characterization of these complex but well-known mechanisms was not the aim of the current study. Instead, the *in vitro* responses of human “non-clinical grade” islets obtained from diverse, non-diabetic human and canine donors (n = 6 each) were investigated and demonstrated that islet gene expression profiles in both species (insulin, others) closely correlate with population doublings and Glucose Sensitive Insulin Secretion (GSIS) responses. Importantly, the degree of *in vitro* de-differentiation and simultaneous expansion levels of ICs must be limited to a point where effective *in vivo* re-differentiation and resumption of physiological hormone production and secretion will occur, as indicated by the remaining gene expression levels for insulin, glucagon, somatostatin and PPY. This collective gene expression profile, obtained by rtPCR, is used as a release criterion of expanded islet cells and their coaggregation with MSCs to from functional NIs.

Importantly, there are well documented phenotypical donor and islet quality differences that have been demonstrated to correlate with the outcome of islet and pancreas transplants. In order to account for such differences and to maximize the possibility of successful outcomes with as few donors as possible, as well as to conserve resources, guidelines and screening systems such as the North American Islet Donor Score (NAIDS) for both donors and islets are now utilized [36–41].

The NAIDS system scores donors based on a number of characteristics including body weight, body surface area, body mass index, use of vasopressors, certain blood chemistries, age, and HbA1c levels [38]. Other studies have demonstrated that human islet viability, purity, size, morphology, and integrity correlate well with *in vivo* function, with viability > 96%, islet diameter of >200 μm for more than 10% of the islets, a solid, spherical shape, and a well-rounded border being highly predictive of a successful clinical transplant outcome, also pretested in STZ-diabetic NOD/SCID mice [41, 42]. However, even with the application of such stringent islet donor quality criteria only a sufficient islet yield for islet transplants is reported by some centers in the range of 30–50% [38]. Nevertheless, the use of the NAIDS and other scoring criteria has the capacity to facilitate the a priori avoidance of actually harvesting islets from suboptimal donors, which is cost and time saving [38, 39, 43–45]. However, since still significant numbers of islet preparations fail to meet the quality criteria that such scoring systems apply, it is inevitable that such “suboptimal” islet preparations may be discarded and thus may potentially represent waste of a highly valuable resource if found to be effective for novel cell-based therapies of T1DM that rely on culture expanded islet cells rather than whole, fresh islets.

Whether such donor and islet differences that are important to islet transplant outcome apply to NI therapy and translate into successful NI-therapy outcomes has not been examined. As this therapy is fundamentally different from islet or pancreas transplants in that the islets are subjected to culture expansion and undergo several doublings prior to incorporation into NIs, there is an argument to be made that such criteria are not relevant to NI therapy outcomes, and that other characteristics of culture expanded islet cells should be used as markers of potency for NI therapy instead. Indeed we note and as shown below the majority of human and canine islets used for our successful NI therapy of both STZ-diabetic NOD/SCID mice and in diabetic dogs would have scored poorly using the above discussed criteria.

In our preclinical mouse and clinical dog studies, we have used residual insulin and other islet hormone gene expression levels as markers of potency and as indicators of the potential of ICs in NIs to re-differentiate into islet-specific endocrine cells when implanted [12]. While therapeutic doses of canine Neo-Islets (cNIs) used to treat both STZ-diabetic NOD/SCID mice and insulin-dependent dogs were formed from IC banks expanded from different canine islet donors, we have thus far not seen a significant, donor-dependent difference in their therapeutic efficacy [12, 14]. Similarly, islets from diverse human donors have been used to form human Neo-Islets (hNIs) for treatment of STZ-diabetic NOD/SCID mice, and donor-dependent differences in therapeutic efficacy have not been observed in those studies, either [13].

Accordingly, the current *in vitro* studies were conducted in order to further demonstrate how the utilized canine and human islets, despite their suboptimal quality scores for islet transplantation [38, 41, 42], functioned well in our NI therapy in both STZ-diabetic NOD/SCID mice and diabetic dogs [12–14], respectively.

For these reasons, the well-known donor- and islet isolation-dependent functional variabilities of freshly harvested human islets [36–39, 43–45] were systematically re-examined after processing them for the production of therapeutically highly effective NIs [12], the 3-D co-aggregates of allogeneic ICs and MSCs [12–14]. Importantly, should permanent and significant post-processing variabilities in the functional characteristics of passaged hICs and cICs be detected, this would be a significant confounding issue in planned human clinical trials in which manufactured NIs from diverse donors are tested. However, the here undertaken detailed analysis of this issue in both processed human and dog ICs did not reveal significant differences in their functional and gene expression profiles, which, in turn, indicates that the consistent and reproducible post-processing functional profiles of tested human ICs significantly facilitates the safe and efficient conduct of a planned human Type 1 Diabetes Clinical Trial. In addition, the *in vivo* therapeutic efficacy of diverse hICs, processed to form human NIs, in STZ-diabetic NOD/SCID mice, as was previously shown with canine NIs, has been confirmed [12, 13]. Of note, in this context, is the well justified and highly relevant conduct of preclinical studies in companion animals such as dogs for human clinical trials, as has been done here [46].

Taken together, the present data in human ICs are paralleled by those in canine ICs, observations that both support the continuation of the ongoing Pilot Study in insulin-dependent dogs [14] and the IND work for the planned human Clinical Trial. Importantly, the NI technology, as demonstrated here, has the potential to greatly reduce the waste of lower quality islets that are judged to be unsuited for a successful islet allotransplant [36–39, 43–45]. Furthermore, the expected therapeutic success with hNIs in Clinical Trials, using the current technology, should translate into a greatly increased number of patients with T1DM that might be functionally cured with this novel form of biotherapy.

## Materials and methods

### Cell culture

#### Islet cell culture

Research grade human islets from 6 non-diabetic human donors (see **Table 1** for demographics and Islet characteristics) were purchased in lots of ~5,000 Islet Equivalents from Prodo Labs (Aliso Viejo, CA). Islet cells derived from this inhomogeneous group of islet donors were expanded by culturing whole islets in tissue culture flasks, using RPMI 1640 medium (Gibco, Thermo Fisher Scientific, Waltham, MA) + 10% human Platelet Lysate (hPL; Cell Therapy and Regenerative Medicine, University of Utah, Salt Lake City) + 1 x L-Glutamine-Penicillin-Streptomycin solution (GPS; Sigma G1146) until ~90% confluent. For passaging, cells were released with 2x Trypsin (Sigma, St. Louis, MO), pelleted by centrifugation at 600x g for 5 min., washed with DMEM 5 mM glucose (Gibco) + 10% hPL + GPS (complete medium), and reseeded at a density of 2x10e5 cells into T75 flasks in complete medium. hICs were characterized by rtPCR for expression of IC specific genes. Human ICs (hICs) and canine ICs (cICs) doubling times and population doublings (PDLs) were calculated by standard methods (see below).

**Table 1:** Demographic and Islet Characteristics of human Islet Donors.

Donor #	Age [yrs]	Gender	Race	Islet Diameter [% >200 μm]	Purity of Islets [%]	BMI [kg/m^2^]	Viability of Islets [%]
**1**	27	Male	Native American	1.5	90	25.8	80
**2**	28	Male	Caucasian	7	95	34.7	80
**3**	40	Male	Hispanic	24.6	90	25.3	70
**4**	48	Female	Asian	8	90	21.5	90
**5**	29	Male	Hispanic	13.4	85	22.8	95
**6**	61	Female	Hispanic	8	85	28.9	90

yrs = years

#### Calculations

Population Doublings (PDLs) = Log(fold)/Log(2), where fold = number of cells at harvest/number of cells seeded. Doubling times = the number of hours in culture/PDL. Endocrine gene and protein expression levels were plotted as a function of PDLs.

#### MSC culture

Human, bone marrow derived MSCs were purchased pre-characterized (tri-lineage differentiation, HLA antigens and surface CD markers) from Lonza (Walkersville, MD) and cultured in complete medium as previously described [12, 47, 48], and used at Passage 3 for formation of NIs.

#### Dog islets and cell lines

Utilized dog islets and Adipose Stem Cells (ASCs) from inguinal fat were identical to those used in our ongoing pilot study (INAD 012–776) and all dog cell lines were cultured as previously described [14]. All donor dogs were non-diabetic mongrels, and 5 of 6 had pacemaker-induced congestive heart failure (see **Table 2** for details). Cadaveric islets and adipose tissue were obtained through an NIH Organ Sharing Agreement at the University of Utah.

**Table 2:** Demographic characteristics of canine pancreas/islet donors and approximate purity and viability of islets (cause of death in all donors was euthanasia; all donors were mongrels and non-diabetic).

Donor #	Age [yrs]	Gender	Health	Purity of Islets [%]	Weight [kg]	Viability of Islets [%]
**1**	1.3	Female	CHF*	50	25	60
**2**	1.3	Male	healthy	95	27	95
**3**	1.3	Male	CHF	90	28.8	85
**4**	1.5	Male	CHF	50	32	95
**5**	3	Female	CHF	80	29	90
**6**	1.5	Female	CHF	70	40	90

*, CHF = Pacemaker-induced Congestive Heart Failure. ^†^, ND = not determined; yrs = years.

### Viability

Islet and cell viability were assessed using Fluorescein diacetate (FDA, Sigma F7378) and Propidium Iodide (PI, Life Technologies P3566) staining, following instructions of the respective manufacturers. Islet viability in percent was quantified in 10 different, homogeneously distributed fields of ~ 400 human and canine islets, respectively. This method does not detect potential apoptotic cell loss (see images in **S1 File**).

### Neo-Islet (NI) formation

NIs were formed as previously reported [12–14]. In brief, human MSCs and canine ASCs and culture-expanded respective ICs were seeded at a density of 0.6–0.8 million total cells per ml of medium. The cells were co-cultured in complete medium at a 1:1 ratio in ultra-low adhesion surface culture dishes (Corning, Kennebunk, ME) overnight, without changing medium, resulting in highly efficient hNI formation.

### rtPCR

RNA was extracted from 1x10e6 cells (Qiagen RNeasy Mini Kit, Germantown, MD). Reverse transcription was performed in duplicate using SuperScript II Reverse Transcriptase for 60 min. at 42°C and species-specific TaqMan primers (Applied Biosystems, Foster City, CA; see **Table 3**) and the ABS 7500 Real Time PCR System (Applied Biosciences, Foster City, CA). All reactions were carried out in a total volume of 20 μL with TaqMan Universal Master Mix II with uracil-N-glycosylase (UNG). Reaction conditions were 50°C for 2 min., followed by a 10 min. start at 95°C, and 40 cycles of melting at 95°C for 15 sec. and annealing at 60°C for 1 min. The average threshold cycle (Ct) value was used for calculations of changes in gene expression. Relative Quantification (RQ), defined as is standard as 2-ΔΔCT where CT is the Cycle Threshold [49] was calculated using the rtPCR machine’s software and through normalization to two internal controls, beta actin (ACTB) and beta 2 microglobulin (B2M). Where indicated, results are presented as Log10 Relative Quantification (RQ, where RQ = 2^-ΔΔCt^). Differences greater or less than log10(RQ) 2 or -2 were considered significant [50].

**Table 3:** rtPCR primers used to test human and dog target gene expression.

Target genes	Applied Biosystems catalog #
***ACTB* (human)**	Hs01060665_g1
***B2M* (human)**	Hs00984230_m1
***INS* (human)**	Hs02741908_m1
***GCG* (human)**	Hs01031536_m1
***SST* (human)**	Hs00356144_m1
***PPY* (human)**	Hs00358111_g1
***ACTB* (dog)**	Cf03023880_g1
***B2M* (dog)**	Cf02659077_m1
***INS* (dog)**	Cf02647520_m1
***GCG* (dog)**	Cf02624195_m1
***SST* (dog)**	Cf02625293_m1
***PPY* (dog)**	Cf02653446_g1

### Glucose Sensitive Insulin Secretion (GSIS)

GSIS was tested on human islets and cultured islet cells as previously described (12,13). In brief, 1,000 islets or 1x10e6 culture expanded islet cells were incubated in 1 ml DMEM 5 mM glucose at 37°C for 1 hour, after which the cells or islets were collected, and the supernatants discarded. The cells or islets were resuspended in 1 ml DMEM 5mM glucose and incubated again at 37°C for 1 hour, after which the supernatant was collected and stored at -20°C until ready for analysis, and the medium was replaced with 1 ml DMEM 25mM glucose. Cells were once again incubated at 37°C for 1 hour, after which this high glucose supernatant was collected and stored at -20°C until ready for analysis. The high and low glucose supernatants were assessed for insulin content using an ELISA kit and following the manufacturer’s instructions (Crystal Chem 90050).

### Statistical analysis

With the exception of rtPCR results (see above), data are expressed as Mean ± SD. Primary data were collected using Excel (Microsoft, Redmond, WA), and statistical and linear regression analyses were carried out using Prism (GraphPad, San Diego, CA). Two tailed t-tests were used to assess differences between data means. A *P* value of < 0.05 between data means was considered significant. Complete data sets are available upon request.

## Results

It was examined whether demographic differences between human islet donors and the quality of isolated islets can adversely affect the characteristics and formation of hNIs, manufactured by co-aggregation of cultured ICs and MSCs. Accordingly, functional profiles of hICs from 6 demographically different, non-diabetic human donors with different body mass indices and IC viabilities and purities (see details in **Table 1** and islet images and human donor and islet characteristics in **S1 and S2 Files**) were compared to each other by assessing their growth rates, islet-specific endocrine gene expression levels as a function of PDLs, GSIS, and their ability to form NIs when co-cultured with MSCs. Identical tests were carried out with canine ICs and ASCs and obtained results were compared to human cell data. Correlation with PDLs was used because (a) the expansion process and entering of the cell cycle trigger the decrease in endocrine gene and protein expression, (b) this expansion is what allows NI technology to partially overcome the donor shortage issue, (c) predictable production endpoints are critical to manufacture of a product, and (d) it is the endocrine expression capacity of the IC component of NIs that is of critical value in this technology, we examined herein whether there was a consistent and predictable correlation between cell expansion and endocrine gene expression.

### Population Doubling Times (PDT)

**Fig 1:**
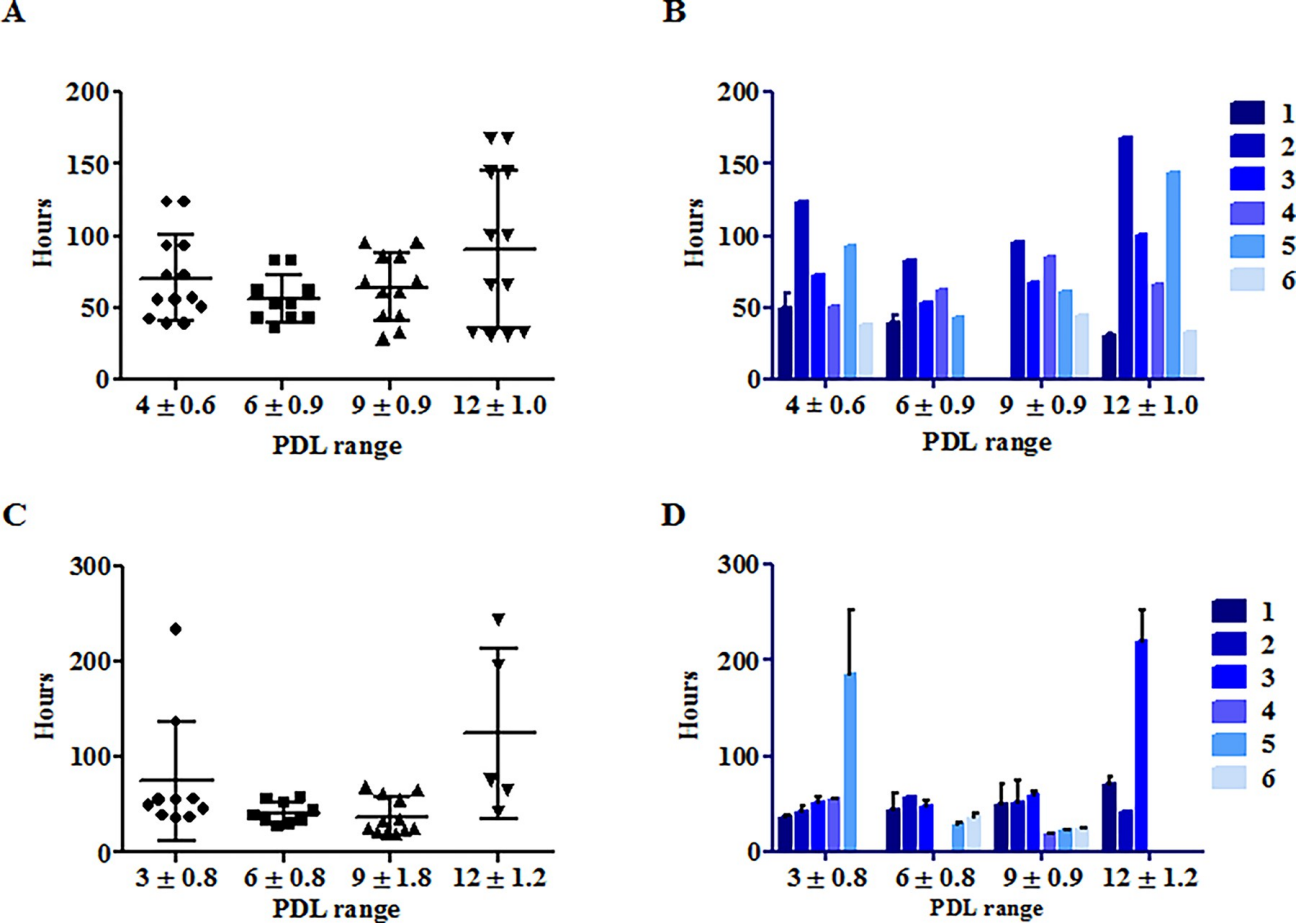
Population Doubling times (PDTs; in hours, Y axis) of human *Islet Cells* (A and B) and canine *Islet Cells* (C and D) as a function of Population doublings (PDLs, X axis) at 3 PDLs (dog cells), 4 PDLs (human cells), and 6, 9 and 12 PDLs (both dog and human cells) (Mean ± SD).

We previously observed that population doublings (PDLs) of cultured mouse and dog ICs between different donors vary significantly, however, this inter-donor variability did not affect subsequent function of either mouse or dog NIs *in vivo* [12, 14]. In the present study, PDTs (Y axis) of hICs and cICs over a range of PDLs (4–12 in human ICs; 3–12 in canine ICs) are shown in **Fig 1**. PDTs ranged from 29 to 170 hrs, with a mean of ~70 hrs. Such variability of doubling times parallels those we previously observed in both mouse and dog ICs [12, 14], and thus is unlikely to be a confounding variable for NI technology.

Doubling times of (**A**) human ICs from the 6 donors listed in **Table 1**, and (**C**) of canine ICs from the 6 donor dogs listed in **Table 2** in hours (Y-axis) over step wise increases in PDLs (X-axis)**)** are shown. Data are presented both grouped (**A** and **C**; mean ± SD) and from individual human (**B**) and dog (**D**) donors. The latter data are shown to better illustrate the striking inter-donor variabilities. All experiments were conducted in duplicate. As we previously reported for mouse and dog ICs [12, 14], human ICs from different donors similarly exhibit significant variations in growth rates.

### Gene expression profiles of insulin and other islet-specific hormones and Glucose Sensitive Insulin Secretion (GSIS) as a Function of Population Doublings (PDLs)

**Fig 2:**
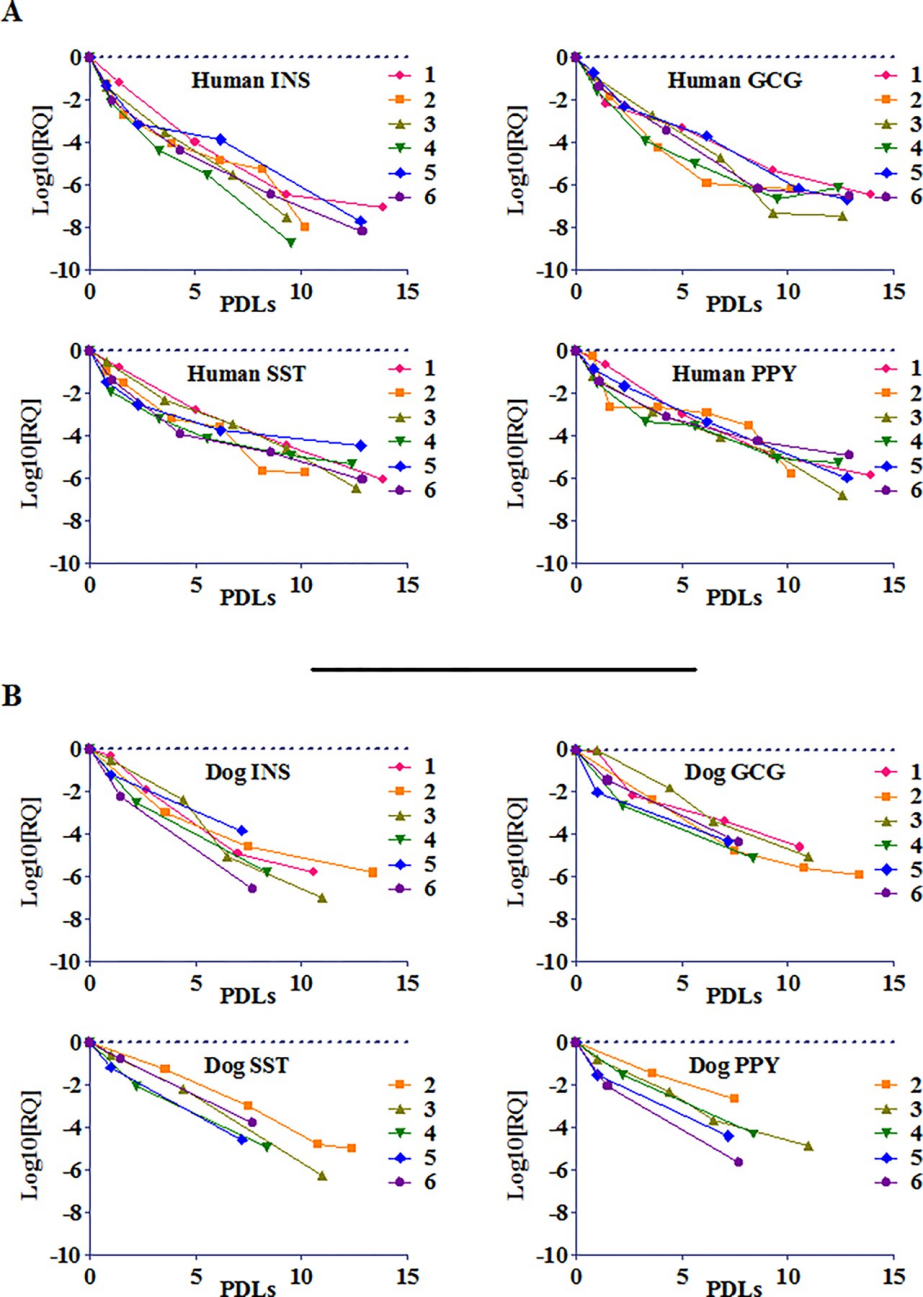
Fold-Change (log10(RQ)) in (A) human and (B) canine ICs’ Endocrine Gene Expression as a Function of Population Doublings (PDLs). Fold change (log10(RQ)) in *INS*, *GCG*, *SST* and *PPY* gene expression (Y axis) as a function of population doublings (PDLs; X axis) for **(A)** human islet donors 1–6, and **(B)** 5–6 representative dog islet donors. For both human and dog islets (points zero on X- and Y-axes), ICs were culture expanded, PDLs determined, RNA isolated, and gene expression assessed as described in Methods. Fold change in gene expression was calculated as log10(RQ) and genes of interest were normalized (ΔΔct) to those of their fresh parent islets (point zero on Y axis), and plotted as a function of PDLs. Culture expanded human ICs are similar to dog cells in that expression of islet-specific endocrine genes decreases linearly as a function of PDLs, and that expression consistently remains detectible for at least 10 cell doublings in human ICs and ~ 7 doublings in dog ICs, respectively. The highly significant r-squared values for the observed linear decrease in gene expression as a function of PDLs are provided in **Table 4**.

For the ongoing NI treatment of diabetic dogs, insulin and other islet hormone gene expression levels are assessed in cultured cICs prior to NI formation, and residual insulin gene expression is used as a measure of *in vivo* potency and serves as release criterion for cICs and cNIs [14]. In order to determine whether hICs (n = 6) and cICs (n = 6) that are being culture expanded per identical protocol are functionally comparable, we systematically assessed in the cell lines from both species the gene expression levels of insulin (INS), glucagon (GCG), somatostatin (SST), and pancreatic polypeptide (PPY) by rtPCR (see **Table 3** for rtPCR primers, and **S1 Table** for gene expression levels) and plotted these as a function of PDLs. As shown in **Fig 2**, for both culture expanded human and dog ICs, the progressive decease in expression levels of INS, GCG, SST, and PPY (Y axis), assessed as a function of PDLs (X axis), followed a parallel pattern and was readily detectible through 5–10 PDLs in hICs and 5–7 PDLs in cICs, and these changes were largely donor independent. Beyond 10 PDLs in hICs donor differences were seen, and beyond 7 PDLs in cICs donor differences were also observed. The progressive decreases in insulin and other islet hormone gene expression levels in culture expanded hICs and cICs were linear (r-squared > 0.91; see **Table 4** for all r-squared values), and these results were comparable to those observed in mouse ICs (**S1A Fig**) and in human ICs when tested for insulin expression levels (**S1C Fig)**. Changes between slopes among the cultured human and canine islet donors were not significantly different for any of the assessed genes and averaged -0.5 +/- 0.09 for assessed human genes, and -0.5 +/- 0.04 for assessed canine genes (**Fig 2**). Similarly, expression levels and, with the exception of dog SST and PPY, extrapolated line elevations were not significantly different from each other between donors. Such linearity of results indicates that culture expansion of either human or dog islet cells will result in a predictable and parallel decrease in islet hormone gene expression, a potential marker of potency, that can be used to determine at what PDL point cell expansion should stop prior to NI formation, as it indicates at what PDL point insulin gene expression will no longer be detectible. Accordingly, the degree of *in vitro* de-differentiation and simultaneous expansion levels of ICs must be limited to a point where effective and timely *in vivo* re-differentiation and resumption of physiological hormone production and secretion will occur. Our extensive analysis of this important issue in culture expanded ICs identified remaining insulin and other islet hormone expression levels that predict effective and timely *in vivo* redifferentiation as 5 PDLs (P1) in mouse and as ~ 10 (P1) in more rapidly proliferating hICs (**S1B and S1D Fig**).

**Table 4:** r-squared values for observed decreases in human and dog endocrine gene expression levels as function of PDLs, normalized to parent islet cells.

	Human				Dog			
Donor	INS	GCG	SST	PPY	INS	GCG	SST	PPY
1	0.9335	0.9363	0.9893	0.9613	0.9637	0.938	ND	ND
2	0.931	0.8742	0.9591	0.8367	0.8998	0.9382	0.9896	0.9942
3	0.9884	0.9598	0.9936	0.9768	0.9722	0.9855	0.9967	0.9737
4	0.9678	0.8349	0.8534	0.8729	0.9635	0.9159	0.9714	0.9915
5	0.9436	0.9776	0.796	0.9886	0.9674	0.8801	0.9868	0.9559
6	0.9517	0.9255	0.9055	0.8966	0.9762	0.9787	1	0.9683
Ave	**0.95267**	**0.91805**	**0.91615**	**0.92215**	**0.95713**	**0.9394**	**0.9889**	**0.97672**

Ave, average; ND, not determined; SST and PPY gene expression levels on one dog cell line were not determined.

In further support of the similarity of cultured IC behavior among species, human and dog ICs, like mouse ICs, have been shown to readily re-differentiate to produce physiologic levels of insulin and other islet hormones *in vivo* [12–14]. Administration of dog NIs to spontaneously diabetic pet dogs significantly reduces blood glucose levels and insulin needs long term, demonstrating that the phenomenon of IC redifferentiation *in vivo* occurs in dogs as well [14]. Finally, when ICs were co-aggregated with MSCs/ASCs to form NIs and administered i.p. to diabetic mice, mouse and dog ICs were shown to redifferentiate *in vivo* and to produce physiologic levels of insulin [12, 14] (see also **S1 Fig parts C** and **D** for human insulin expressing cells). The largely parallel results observed in **Fig 2** for culture-expanded human vs. dog ICs support our hypothesis that the NI technology, already demonstrated to eliminate or reduce the need for insulin in diabetic mice and dogs, respectively, possesses substantial translational promise for its clinical testing and treatment of human T1DM.

**Fig 3:**
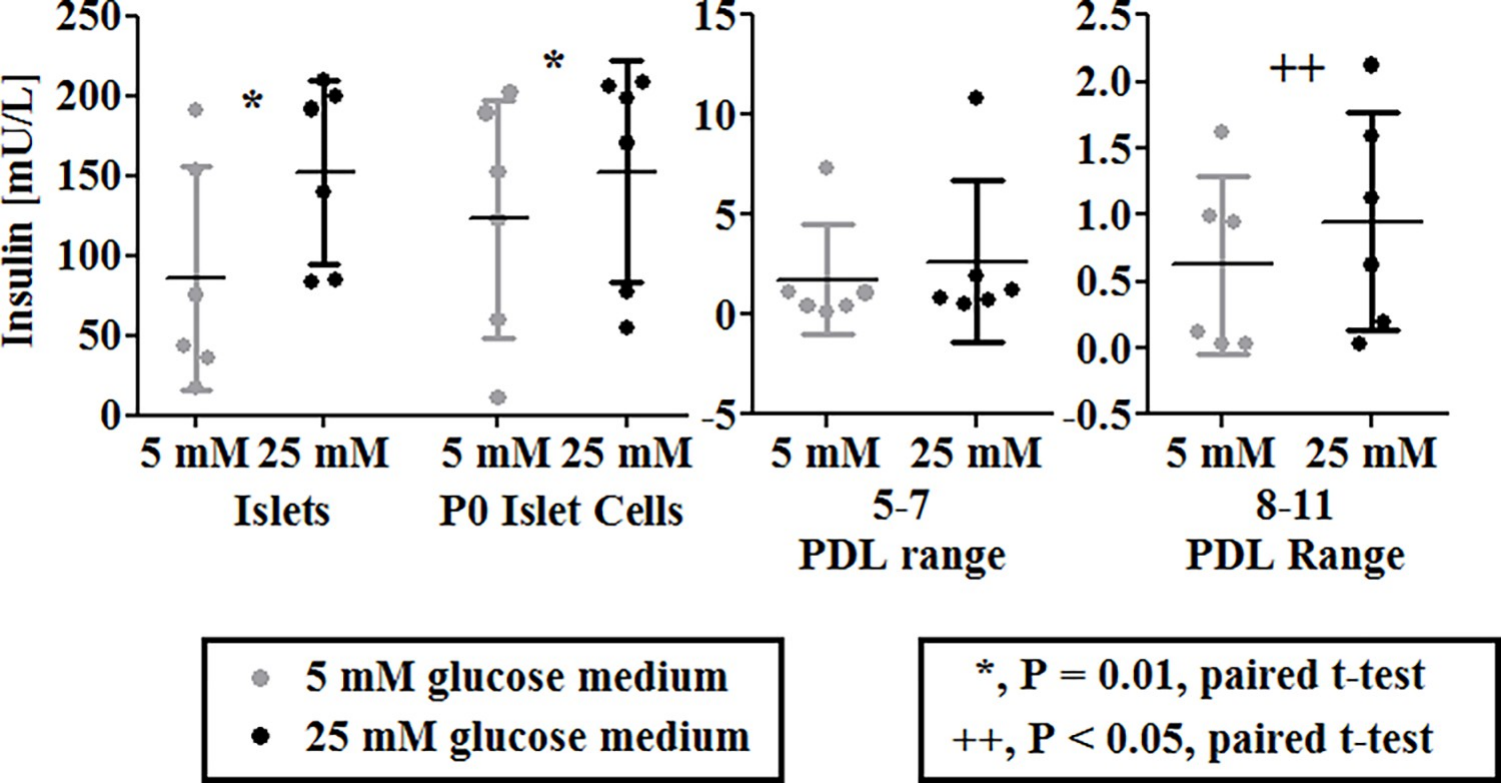
Comparative GSIS of human Islets and cultured human islet cells from 6 donors as a function of PDLs. Summary of GSIS data of human islets and culture expanded hICs (n = 6 each). Insulin secretion (Y axis) to first exposure to 5mM (low) and then 25 mM (high) glucose medium, for 60 min each, was assessed for islets from each donor as well as culture expanded islet cells and plotted as a function of PDLs (X axis). Data are presented as mean ± SD. While the ability to secrete insulin in response to glucose stimulation varies among donors, and culture expansion dramatically reduces the level of insulin secretion, hICs continue to secrete ever decreasing amounts of insulin in response to glucose (see Y axes), similar to what we previously reported for culture expanded mouse and dog ICs [12, 14].

We previously reported that culture expanded mouse and dog ICs and NIs secrete insulin in response to glucose stimulation, albeit at significantly reduced levels compared to freshly isolated, whole islets [12, 14]. Furthermore, dog NIs implanted into STZ diabetic NOD-SCID mice durably induced euglycemia. When retrieved 9 weeks later, these cNIs secreted 15-fold higher concentrations of canine insulin in response to glucose stimulation than did freshly formed dog NIs, clearly demonstrating that they had re-differentiated *in vivo* [14]. To assess whether culture expanded human ICs also secrete insulin in response to glucose, culture expanded hICs from the donors in **Table 1** were tested per GSIS at different passages and their insulin secretion was compared with that of their parent islets (**Fig 3**). As with dog and mouse ICs, culture expanded hICs show glucose stimulated insulin secretion up to 8–11 PDLs, but again at greatly and progressively reduced levels compared to freshly isolated, native islets (**Fig 3**).

### NI formation

**Fig 4:**
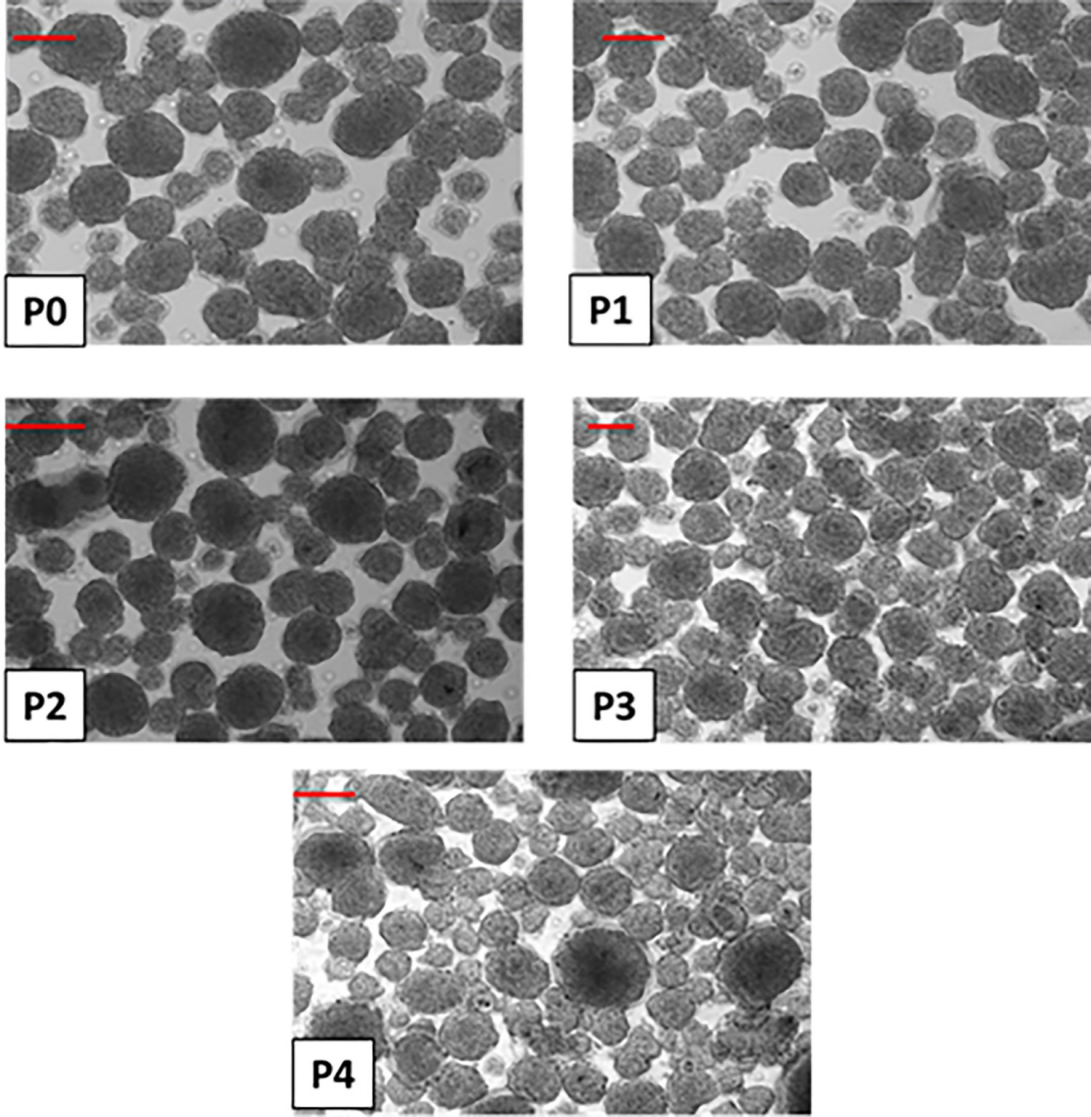
NIs formed by co-culture of human islet cells at passages P0 –P4 and P3 human MSCs. Scale bar (red) = 100 μm. The phenotype of formed hNIs at each tested passage (P0-P4 for ICs only; MSCs were always used at P3) appears comparable. There are only small numbers of single, non-aggregated cells, demonstrating the high efficiency of NI formation, similar to that previously observed for mouse and dog cells [12, 14].

Islet cells from the human donors listed in **Table 1** were tested at passages P0 –P4 for their ability to form NIs when co-cultured with human MSCs (P3) as described in Methods. Islet cells from all donors and from these passages readily and efficiently formed phenotypically comparable NIs both in size and shape similar to those previously observed for both mouse and dog cells (**Fig 4**) [12, 14]. Such results indicate that human donor variations are unlikely to be a significant compounding factor for this aspect of the NI technology.

## Discussion

The present study examined the central question whether donors and their islets that may be judged unsuitable for clinical islet transplants based on the application of the NAIDS and other scoring criteria [37, 38, 41, 45] can instead be used to manufacture NIs that have the capacity to significantly improve insulin needs and glucose levels and to eventually re-establish euglycemia and insulin-independence. Specifically, the impact of demographic differences of human and canine pancreatic islet donors and/or phenotypic variations in islet characteristics such post isolation quality, viability, purity and growth rates were analyzed.

For this purpose, the islets from both 6 diverse human and 6 diverse dog islet donors were analyzed *in vitro* as to their growth characteristics in culture, gene expression profiles (insulin, glucagon, somatostatin, PPY) as a function of their population doubling times. In addition, GSIS and their ability to form NIs with Mesenchymal or Adipose Stromal Cells were examined. Obtained results of culture expanded islet cells were compared to the characteristics of their respective parent cells, i.e., freshly isolated islets.

Unexpectedly, neither dog nor human islet characteristics, as profiled here, revealed any significant differences between the groups of diverse donors. And significantly, the formation of therapeutic quality of NIs from both species was not adversely affected by inter-donor differences in age, cause of death, demographic differences, BMI, islet morphology, purity, or viability [37, 38, 41, 42, 45]. It is of note that the prognostic value of the BMI unlike other donor characteristics remains controversial [37, 38, 45], i.e., a higher BMI of a donor is more recently seen as a positive indicator for a successful islet transplant, and at least a positive indicator of successful islet isolation [36, 41, 43].

Taken together, the primary objective of the present study was therefore to determine whether demographic and islet characteristic differences would adversely affect the biological *in vitro* characteristics of both cultured canine and human ICs used in the production of therapeutic NIs. The data presented here show that the employed processing of canine and human ICs from diverse donors apparently eliminated the potential adverse impact that islet donor variety or islet quality may exert on their essential *in vitro* characteristics. This, in turn, is a highly relevant observation as it demonstrates the clear suitability of islets from diverse donors for the novel cell-based therapy of Type 1 Diabetes with NIs, i.e., islets that other ways may have scored too low for a clinical islet transplant. This important point is further bolstered by the fact that several of the herein examined donor islets have been processed and used to form NIs that have already been shown to be effective in establishing euglycemia in diabetic NOD and NOD/SCID mice, and significantly and durably improving blood glucose levels in diabetic dogs, while eliminating or ameliorating the need for exogenous insulin [12–14].

Of note, and in support of our conclusions, Dog 3 ICs (see Table 2 above) were used to produce a therapeutic dose of NIs and administered to VSH-02 under INAD 012–776, as part of an ongoing study, and as previously described [14]. Insulin gene expression was Ct = 36.9 in the NIs administered to this dog, and the cells had undergone approximately 11 PDLs. VSH-02 was a 6 year old (at time of treatment), neutered male, Bichon mix with a history of hypothyroidism, insulin dependent T1DM (6 months duration at time of treatment–with preexisting islet autoantibodies confirmed by FACS for anti-IC serum IgG), and elevated Alkaline Phosphatase, ALT, Cholesterol, Triglycerides and hyperkalemia prior to treatment. He had no history of pancreatitis, but with evidence on ultrasound of suspected chronic or previous pancreatitis. The screening ultrasound also revealed mild renal degenerative changes. His hypothyroidism was controlled through treatment with 0.1 mg Synthroid BID, and his lipid levels and liver enzymes normalized and were monitored for 6 months prior to NI therapy. Preliminary (6 months follow-up) results were previously published [14]. At that time, he had a significant decrease in HbA1c from 12.1 to 6.8%. This dog has subsequently completed the 3 year follow-up, and had a sustained 41% reduction in the need for insulin (11 U per day to 6.5 U per day) at 36 months post treatment, along with a concomitant reduction in averaged serum glucose by 16.7 mg/dL and serum fructosamine from 574 to 395.2 μM. The islets used for this dose were only ~85% viable, had what would be considered poor morphology, and came from a donor with heart failure (see **Table 2** and **S1 File**), yet the NIs produced from them were effective in significantly reducing blood glucose levels and the need for insulin long term.

The other very significant conclusion from the present studies addresses the rather extreme scarcity of both pancreas and islet donors. Specifically, the expansion of ICs and formation of NIs by their co-aggregation with bone marrow-derived Mesenchymal or Adipose-derived Stromal Cells [12, 14], permits the generation of up to 270 intraperitoneally delivered therapeutic human NI doses from a single donor for ~70 kg recipients with T1DM (See **S1 Fig**), a therapy that does not require the use of potentially toxic anti-rejection drugs or encapsulation devices. In this fashion, the clinical use of this therapy is expected to substantially grow and thereby provide increasing numbers of patients with T1DM with a novel therapy that still needs to undergo the planned and needed testing in an appropriately designed, albeit minimally invasive Clinical Trial. Accordingly, we have obtained approval from the FDA for the conduct of an IND-enabling Proof of Concept study that is currently ongoing.

The NI technology facilitates physiological insulin and other hormone secretion from the omentum, the permanent engraftment site of NIs, into the portal system of the liver, which parallels the delivery pathway of all islet hormones from the pancreas. No adverse or serious adverse events of the NI therapy have so far been observed in NI treated diabetic dogs, now exceeding 3 years of follow-up.

As discussed above regarding the employed cellular mechanisms, the Neo-Islet (NI) technology utilizes the culture expansion of islets, which results in the outgrowth and robust *in vitro* expansion of the major pancreatic islet hormone-producing cells (α, β, γ, δ), a response that is coupled to their expected progressive de-differentiation. This well documented *in vitro* response is simultaneously carried out by serum-induced cell cycle entry and cell proliferation, reversible Epithelial-Mesenchymal Transition (EMT), and by the triggering of the reversible Regenerative Repair Program in sublethally, usually ischemically injured islet cells during islet isolation. Re-differentiation of the cultured islet cells both *in vitro* and *in vivo* occurs by reversal of EMT and exit from the cell cycle upon withdrawal of serum-containing culture media, and completion of the Regenerative Repair Program [27–31]. Of note is the fact that the MSCs in the NIs besides blocking apoptosis, inflammation, fibrosis, adverse actions of Reactive Oxygen Species and auto- and allo-immune attacks on allogeneic cells in the NIs, have the capacity to also support the redifferentiation of the ICs in the NIs, mediated by pro-angiogenic factors and miR-375 release and others mechanisms [19–21, 32–35]. An again, the further investigation of these complex but well-known mechanisms was not the aim of the current study.

Of note is the fact that the starting material, i.e., islets, for human and canine IC expansion and subsequent NI formation came from demographically different donors and corresponding islet viability varied between 60 and 95%. Obviously, low islet viability will reduce the number of ICs for expansion and formation of NIs. However, despite this limitation, we observed that the employed NI generation protocols largely compensated for these differences in islet quality. This clearly demonstrates that potential inter-islet donor variabilities and reduced islet viability do not significantly affect the manufacturing of high numbers of therapeutic NI doses.

## Conclusion

In conclusion, “poor quality” of freshly isolated human and canine islets that may be judged as unsuited for islet transplantation, appear to possess great utility for the manufacturing of high numbers of NIs, 3-D organoids of Mesenchymal Stromal and Islet Cells that have proven to possess excellent therapeutic efficacy in pre-clinical Type 1 Diabetes studies, thereby identifying this novel, minimally invasive, retrievable and economical stem cell-enabled technology as significant progress for the conduct of clinical diabetes trials and eventual therapy. Taken together, we posit that human-NIs created from diverse, “non-clinical grade” donors have the capacity to greatly expand patient access to this curative therapy, facilitated by the efficient *in vitro* expansion of islet cells. Furthermore, biotherapy-specific adjustments in the current donor and islet scoring systems may be warranted. In recognition of our data and in preparation for a subsequent clinical trial, the FDA has approved our IND-enabling Proof of Concept study that is currently ongoing.

## Supporting information

S1 Fig *In vitro* dedifferentiation of cultured murine and human islet cells and their *in vivo* Redifferentiation as the endocrine component of i.p.-administered NIs. (DOCX)

S1 Table Gene expression levels (Cycle Threshold (Ct) and normalized to internal controls (ΔCt)) of dog and human ICs. (XLSX)

S1 File Images of the dog and human islets listed in Tables 1 and 2 prior to culturing, and stained for viability. (PPTX)

S2 File Checklist for reporting human islet preparations used in research. (PDF)

S3 File Data sets for figures. (XLSX)
