# Supplementary material for: Significant expansion of the donor pool achieved by utilizing islets of variable quality in the production of allogeneic “Neo-Islets”, 3-D organoids of Mesenchymal Stromal and islet cells, a novel immune-isolating biotherapy for Type I Diabetes
Source: PLoS One. 2023 Aug 24;18(8):e0290460. doi: 10.1371/journal.pone.0290460 (PMC10449143; doi:10.1371/journal.pone.0290460)
Supplement: S1 Fig — (DOCX) [file pone.0290460.s001.docx]

**S1 Figure**


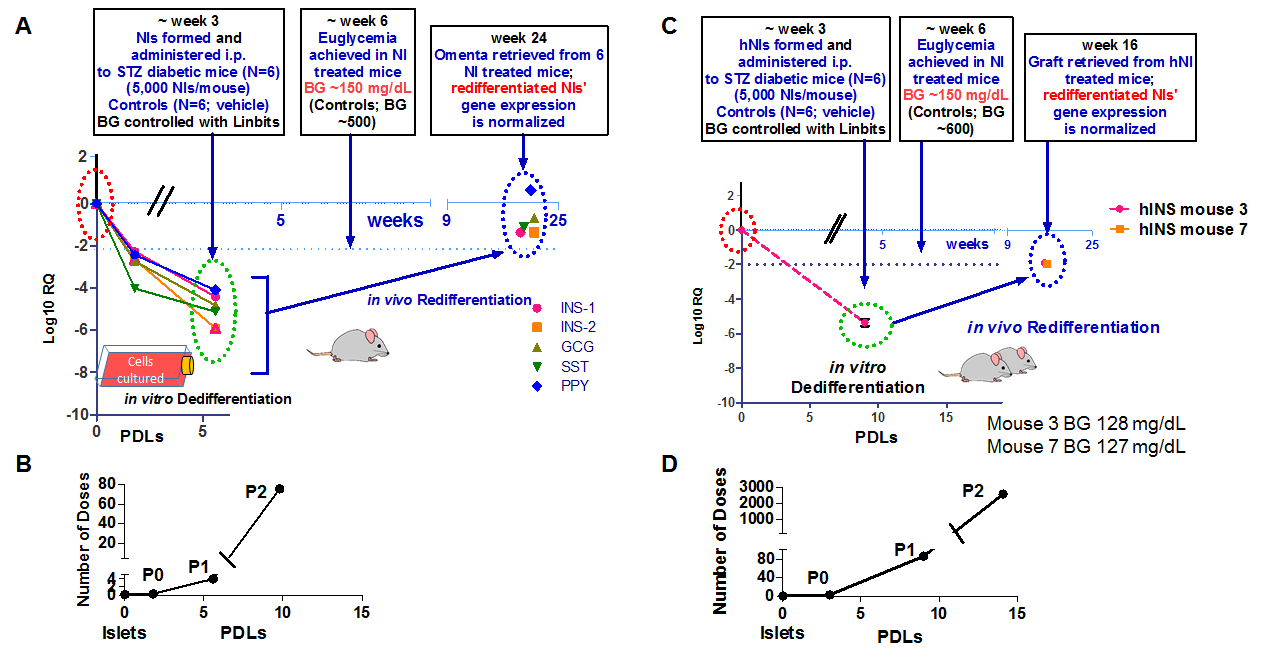


**S1 Figure: *In vitro* Dedifferentiation of cultured murine and human Islet Cells and their *in vivo* Redifferentiation as the endocrine component of i.p.-administered NIs.**

**Panel A**: Dedifferentiation-induced decrease in Islet-specific endocrine gene expression levels of c57Bl/6 mouse ICs normalized to those of whole parent islets and plotted as a function of PDLs *in vitro* and upon NI retrieval from euglycemic STZ diabetic mice at 21 weeks post administration. Mouse islets were isolated, culture expanded, and assessed for islet-specific endocrine hormone gene expression levels by rtPCR as described in Methods. The endocrine gene expression level of whole islets is defined as zero and is found in the red circle. Mouse ICs at ~6 PDLs (~ 3 weeks) were co-aggregated with murine MSCs to from NIs which were used to treat diabetic mice using the previously reported protocol [12]. The endocrine gene expression levels of culture expanded ICs at the time they were cocultured with MSCs to form NIs is found in the green circle. At 21 weeks post i.p. treatment of STZ-diabetic mice (n=6) with NIs (5,000/kg b.wt.; persistent euglycemia was achieved by week 6), gene expression levels of NIs retrieved from the mouse omenta were normalized to expression levels of freshly isolated islets (see blue circle), indicating effective *in vivo* redifferentiation (see arrow and symbols representing examined genes). These data demonstrate that although culture expansion of islet cells decreases endocrine gene expression as a function of PDLs, incorporation of ICs into NIs and implantation into a diabetic subject result in redifferentiation of the ICs, reestablishment of euglycemia, and restoration of islet hormone gene expression.

**Panel B:** This Fig. complements the data in **panel A**, showing the number of NI doses that can be generated from cultured murine ICs at P0-P2 (Y axis) and up to 10 PDLs (X axis). As shown in **panel A**, NIs were generated from ICs that have undergone 5 PDLs, i.e., at a culture expansion point where complete *in vivo* redifferentiation and resumption of function of the islet hormone-expressing cells does occur and that results in the animals’ normoglycemia. As shown in **Fig. 4** above, the phenotype and quality of NIs (size, integrity) up to passage 4 remains stable. However, for the shown experiments in **S1 A-C**, the number of IC passages was set at P1.

**Panel C:** This Fig. shows that human NIs that were generated from culture expanded hICs with reduced insulin expression and human MSCs underwent, post i.p. administration, *in vivo* redifferentiation that resulted in normoglycemia in STZ-diabetic NOD/SCID mice (n=2), an identical therapeutic effect shown in **panel A** animals and as we previously reported [13].

**Panel D:** This Fig. complements the data in **panel C**, showing the number of NI doses that can be generated from cultured human ICs at P0-P2 (Y axis) and up to 15 PDLs (X axis). As shown in panel C, NIs were generated from hICs that have undergone 10 PDLs, i.e., at a culture expansion point where complete and timely *in vivo* redifferentiation and resumption of function of the islet hormone-expressing cells does occur and that results in the animals’ normoglycemia. As shown in **Fig. 4** above, the phenotype and quality of NIs (size, integrity) up to passage 4 remains stable. However, for the shown experiments shown in **S1 Figures A-D**, the number of IC passages was set at P1.

The following mouse primers for experiments were obtained from ABS.

| **Target genes** | **Applied Biosystems catalog #** |
| --- | --- |
| *Actb* | Mm04394036_g1 |
| *B2m* | Mm00437762_m1 |
| *Ins1* | Mm01259683_g1 |
| *Ins2* | Mm00731595_gH |
| *Gcg* | Mm01269055_m1 |
| *Sst* | Mm00436671_m1 |
| *Ppy* | Mm01250509_g1 |
