# Supplementary material for: Significant expansion of the donor pool achieved by utilizing islets of variable quality in the production of allogeneic “Neo-Islets”, 3-D organoids of Mesenchymal Stromal and islet cells, a novel immune-isolating biotherapy for Type I Diabetes
Source: PLoS One. 2023 Aug 24;18(8):e0290460. doi: 10.1371/journal.pone.0290460 (PMC10449143; doi:10.1371/journal.pone.0290460)
Supplement: S1 File — (PPTX) [file pone.0290460.s003.pptx]

## Slide 1
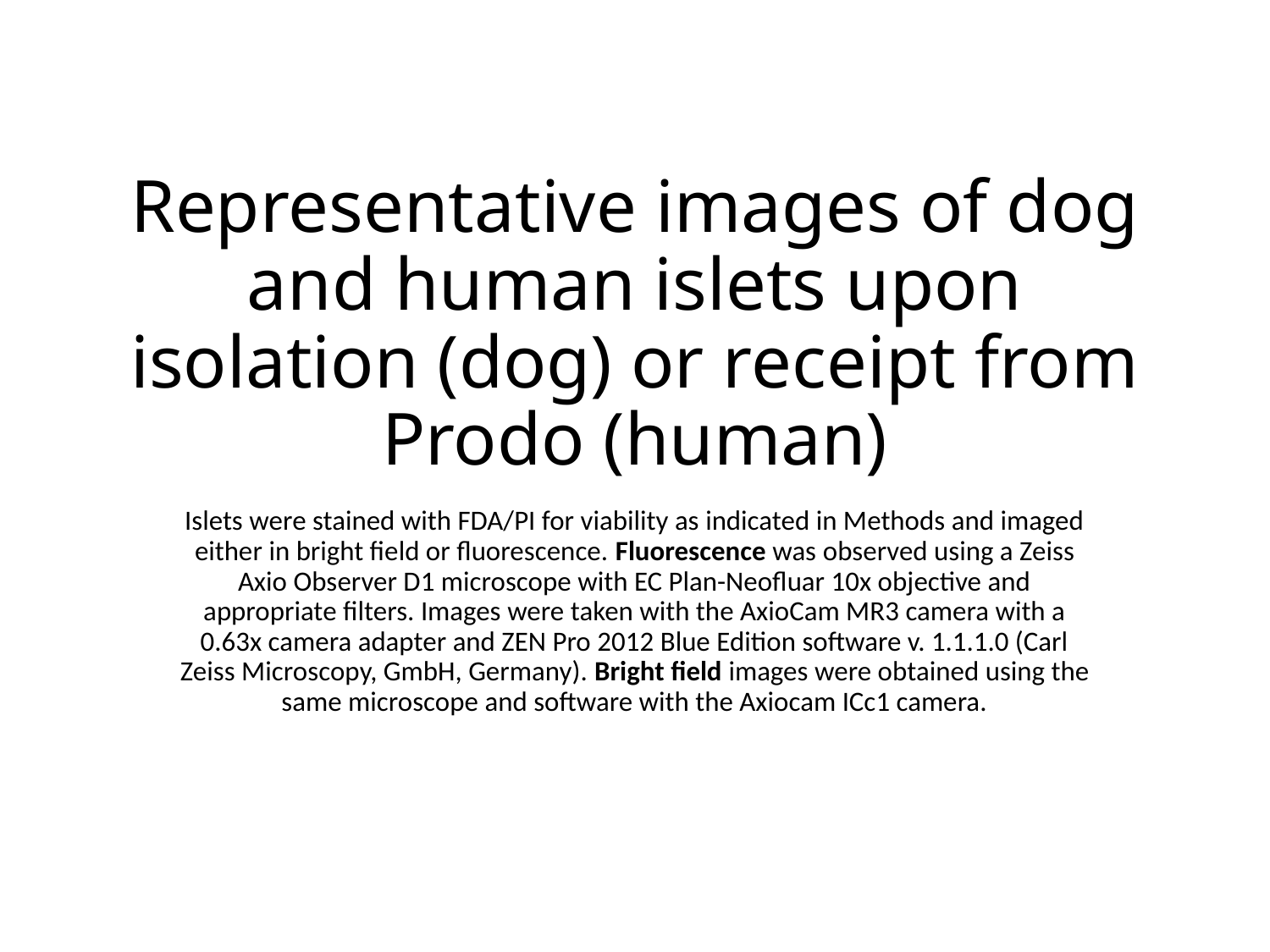

# Representative images of dog and human islets upon isolation (dog) or receipt from Prodo (human)
Islets were stained with FDA/PI for viability as indicated in Methods and imaged either in bright field or fluorescence. Fluorescence was observed using a Zeiss Axio Observer D1 microscope with EC Plan-Neofluar 10x objective and appropriate filters. Images were taken with the AxioCam MR3 camera with a 0.63x camera adapter and ZEN Pro 2012 Blue Edition software v. 1.1.1.0 (Carl Zeiss Microscopy, GmbH, Germany). Bright field images were obtained using the same microscope and software with the Axiocam ICc1 camera.

## Slide 2
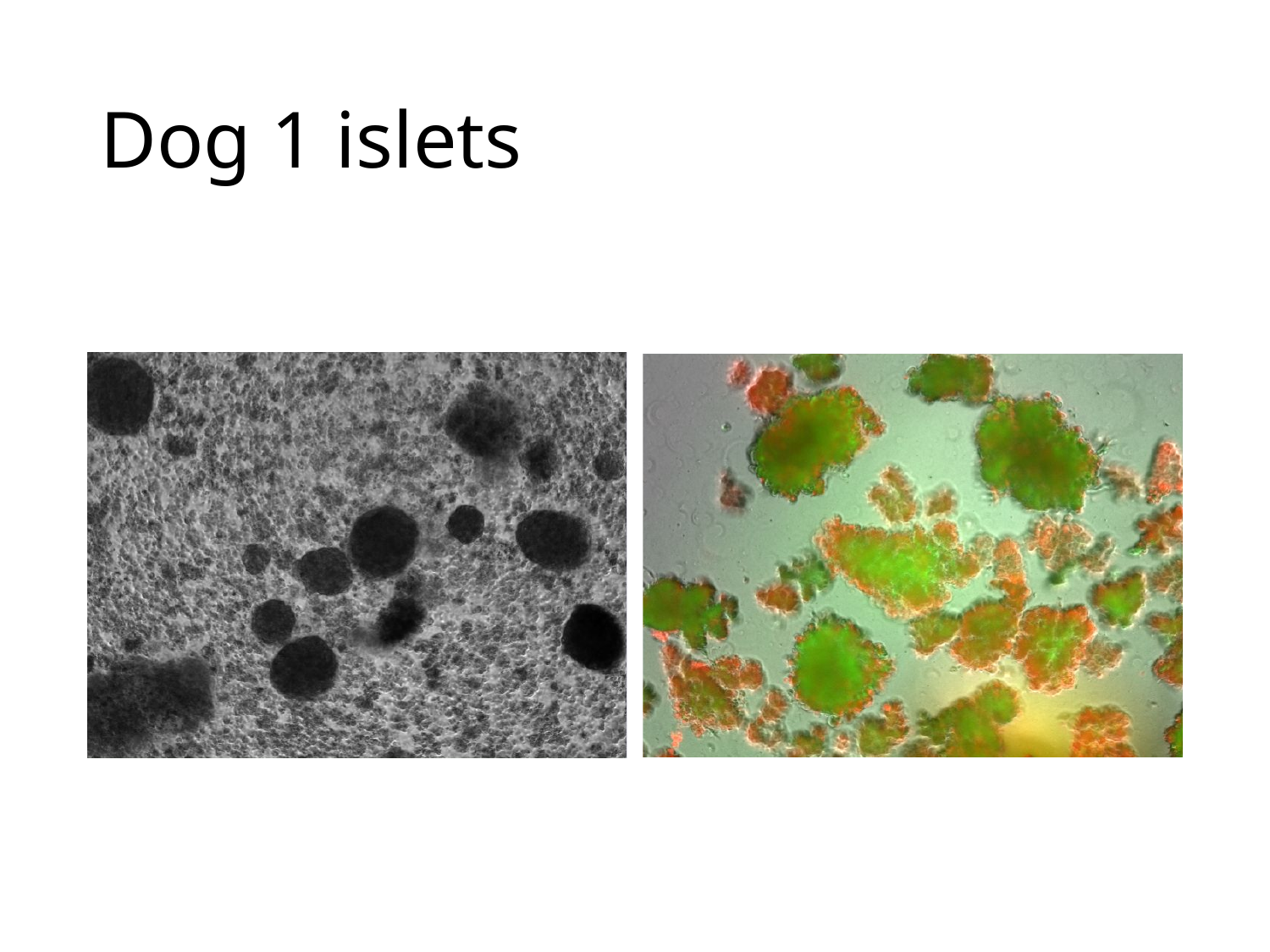

# Dog 1 islets

## Slide 3
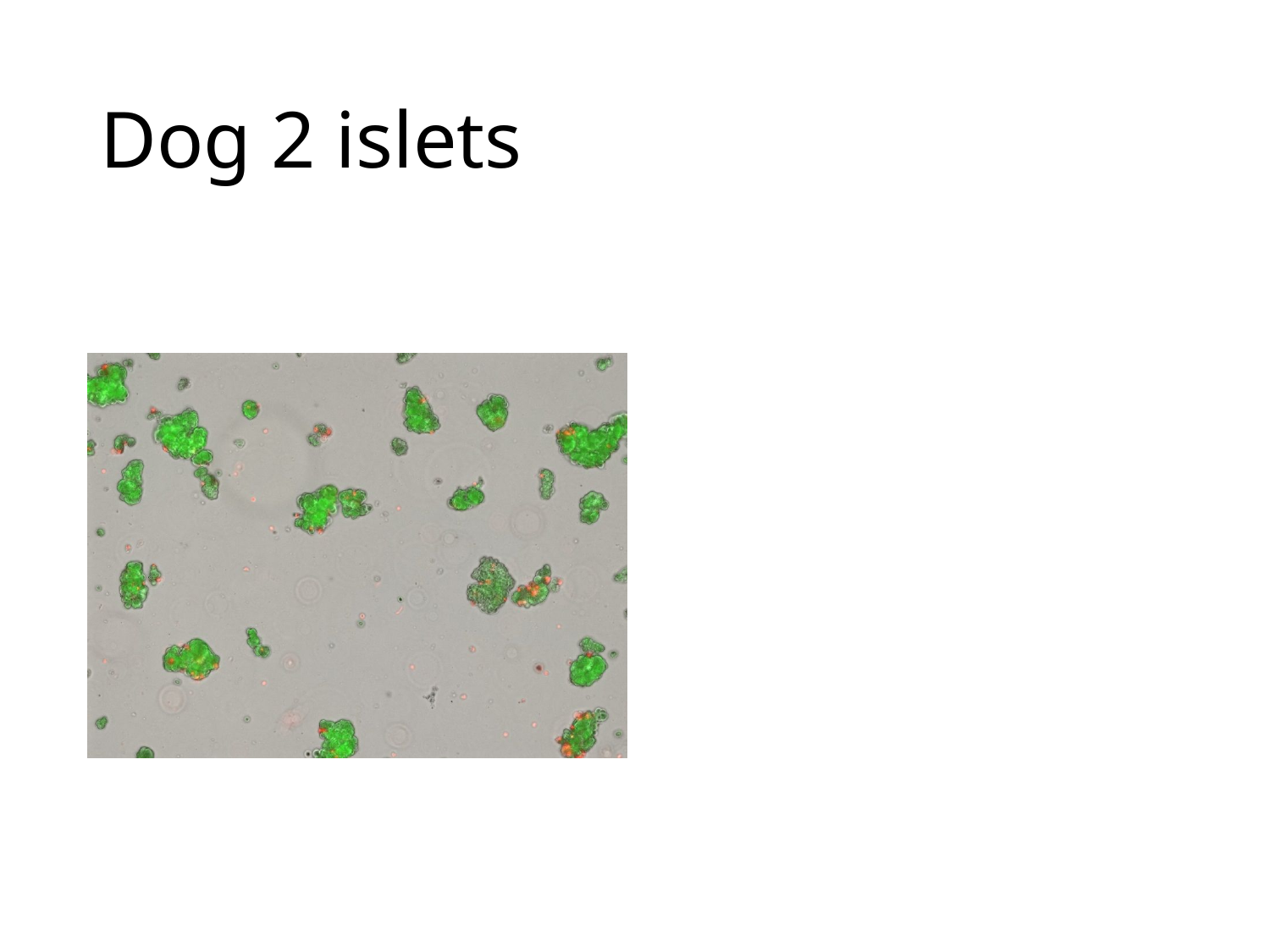

# Dog 2 islets

## Slide 4
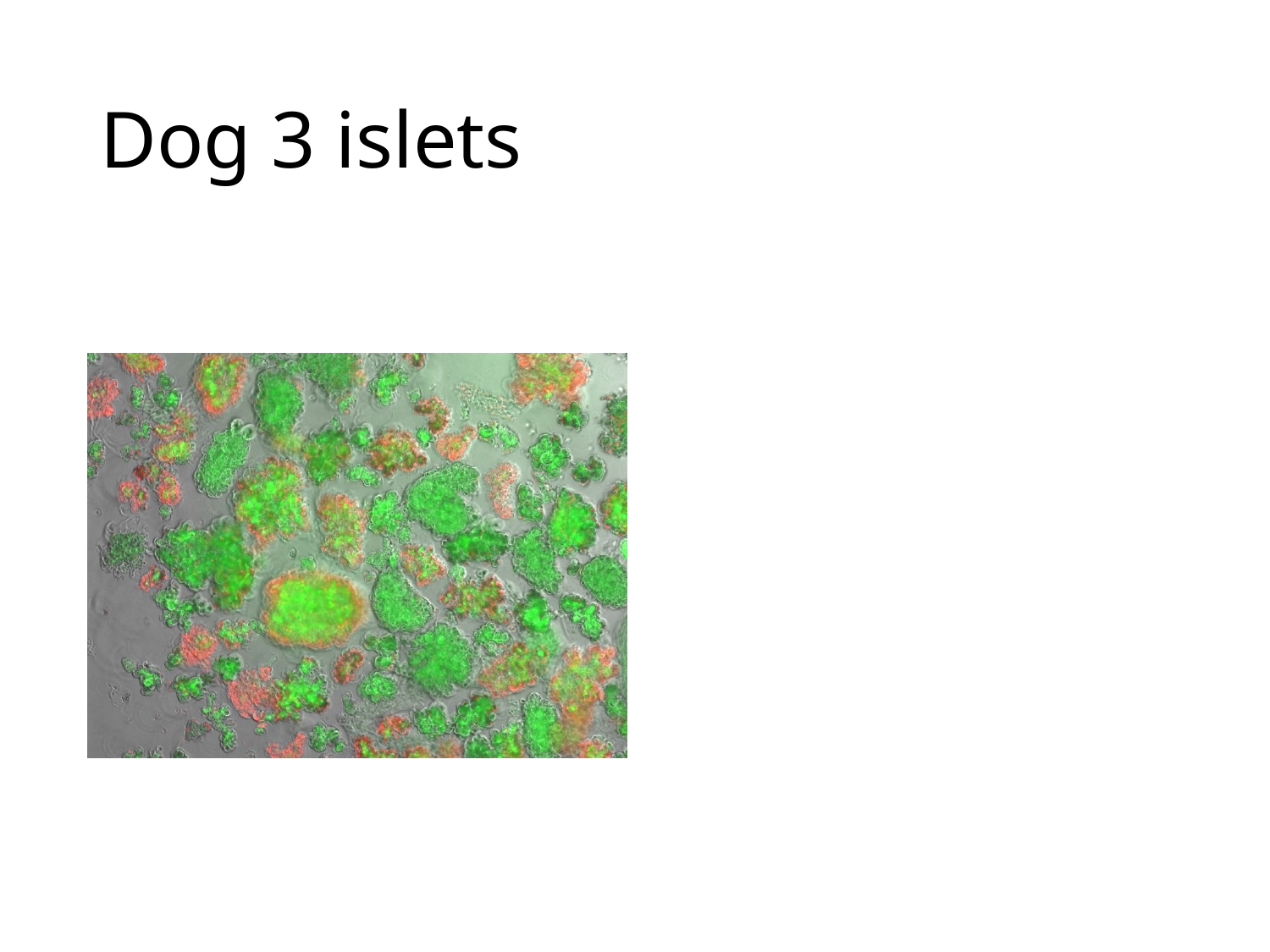

# Dog 3 islets

## Slide 5
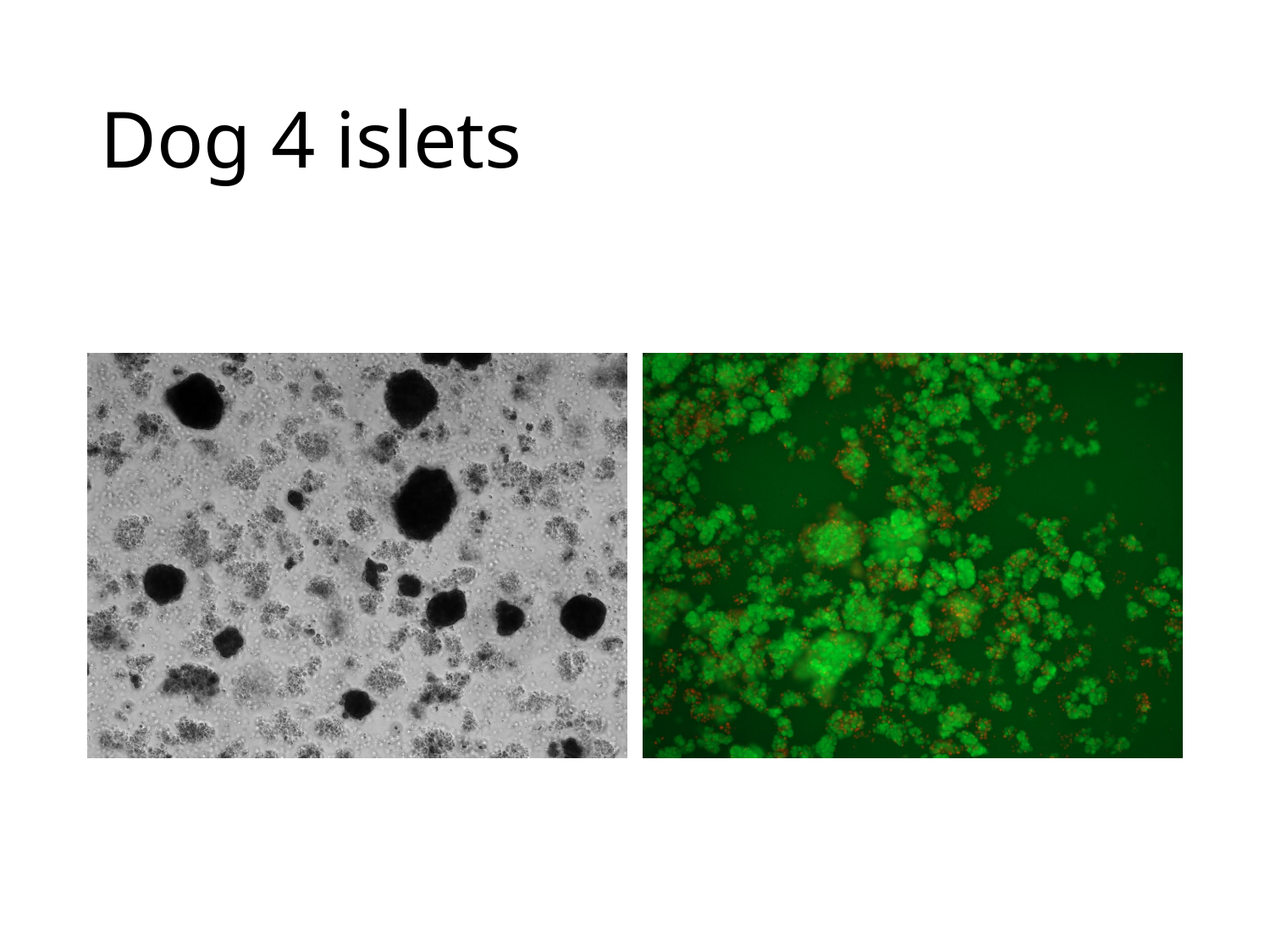

# Dog 4 islets

## Slide 6
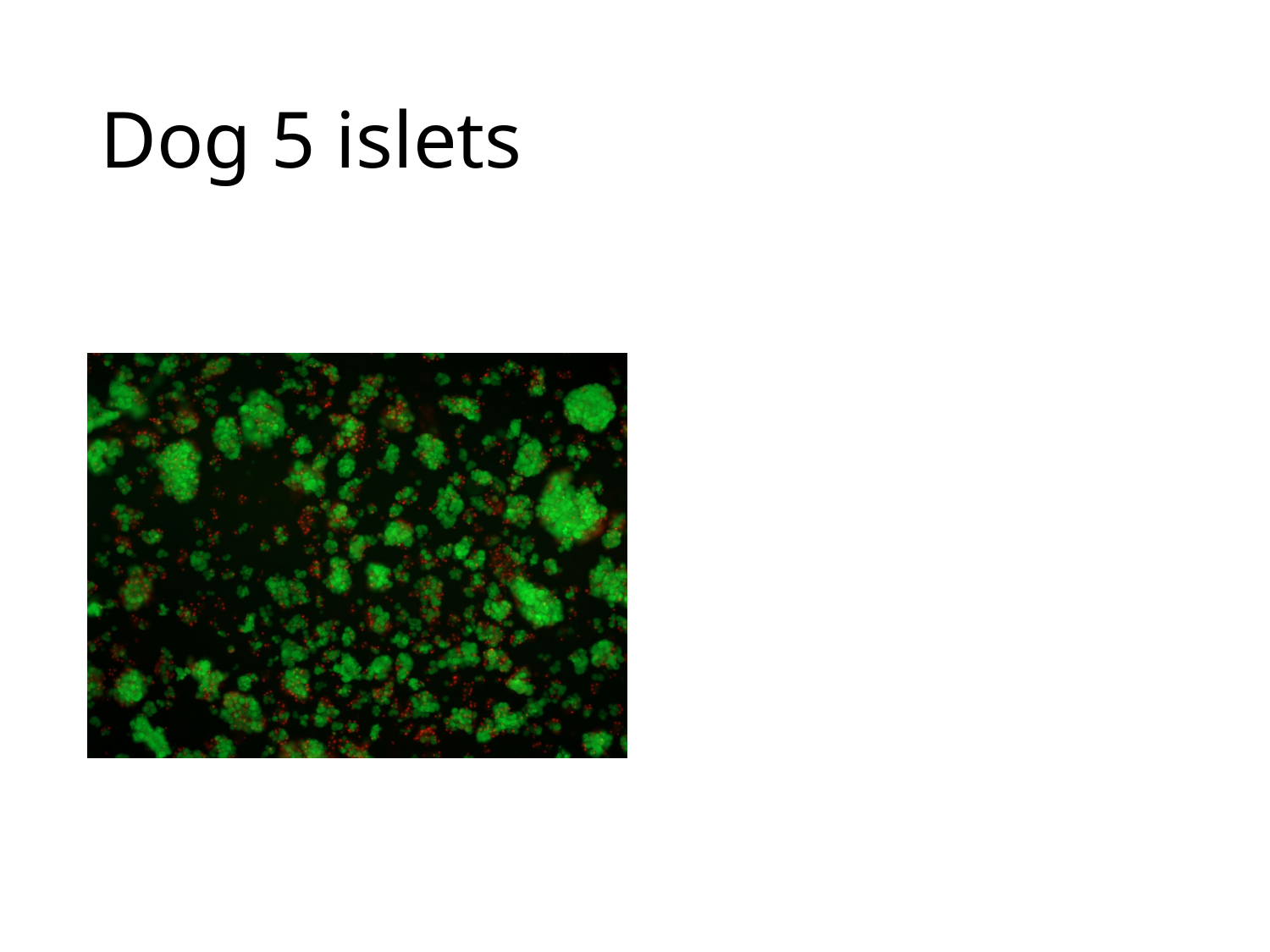

# Dog 5 islets

## Slide 7
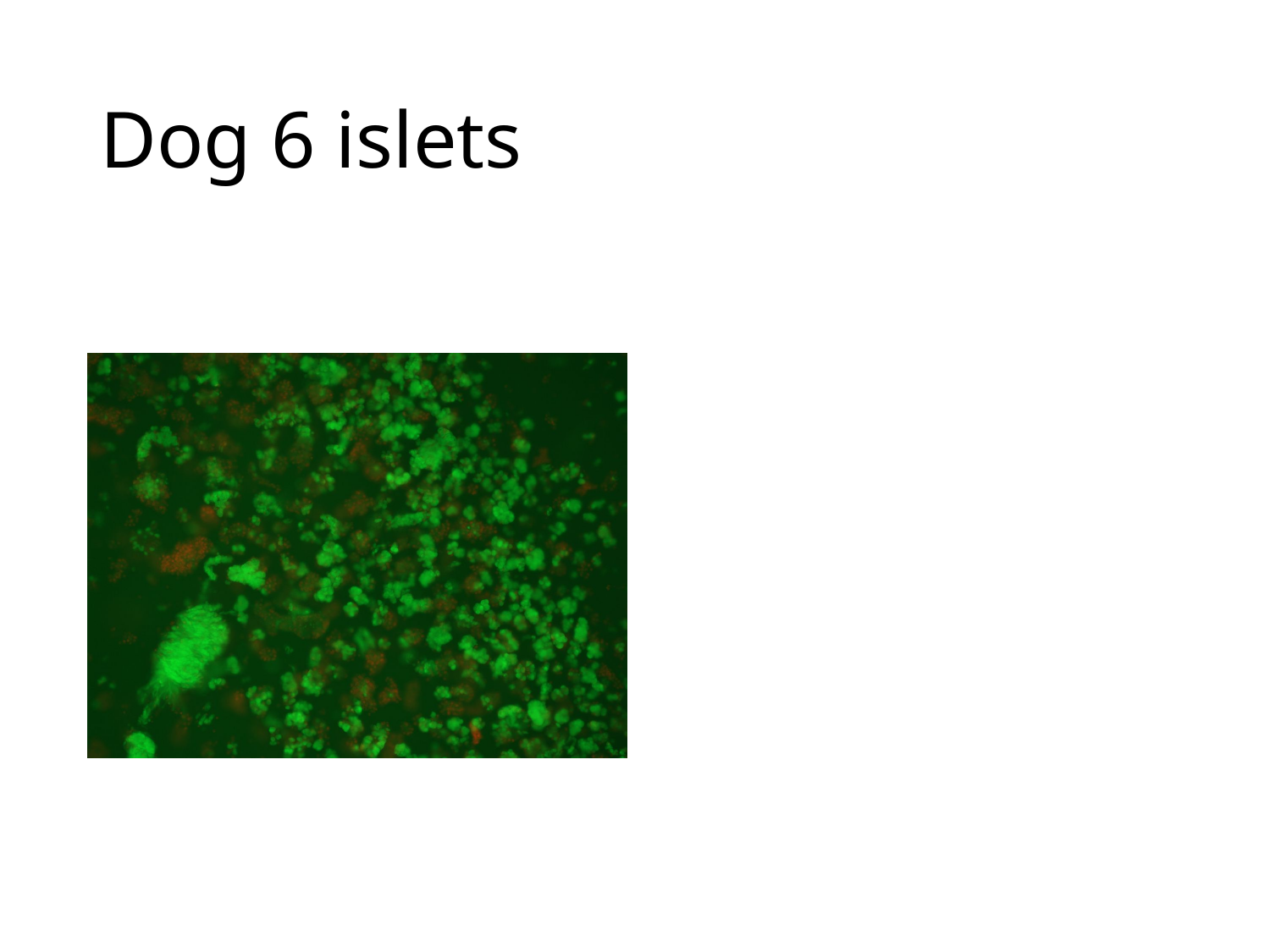

# Dog 6 islets

## Slide 8
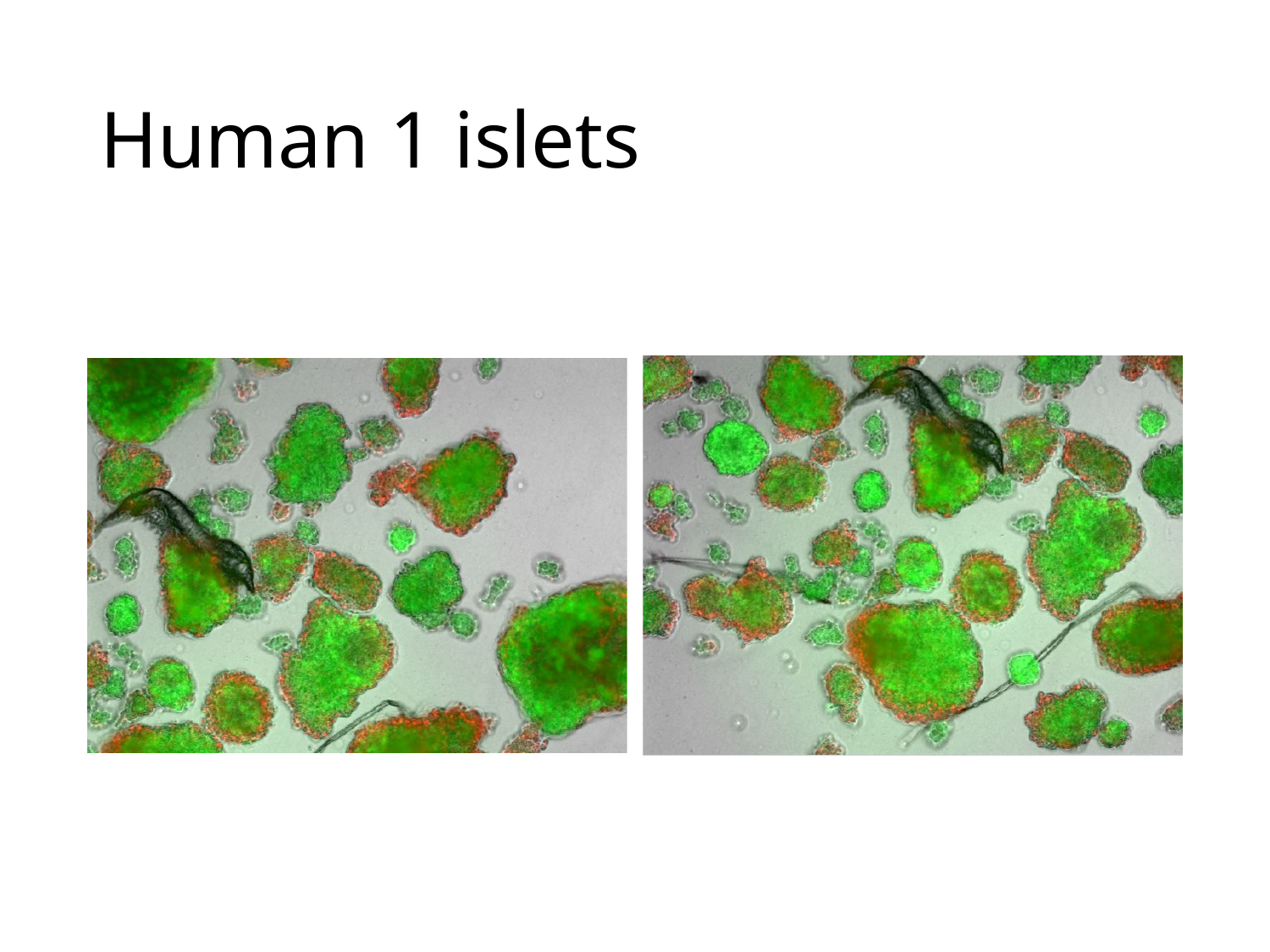

# Human 1 islets

## Slide 9
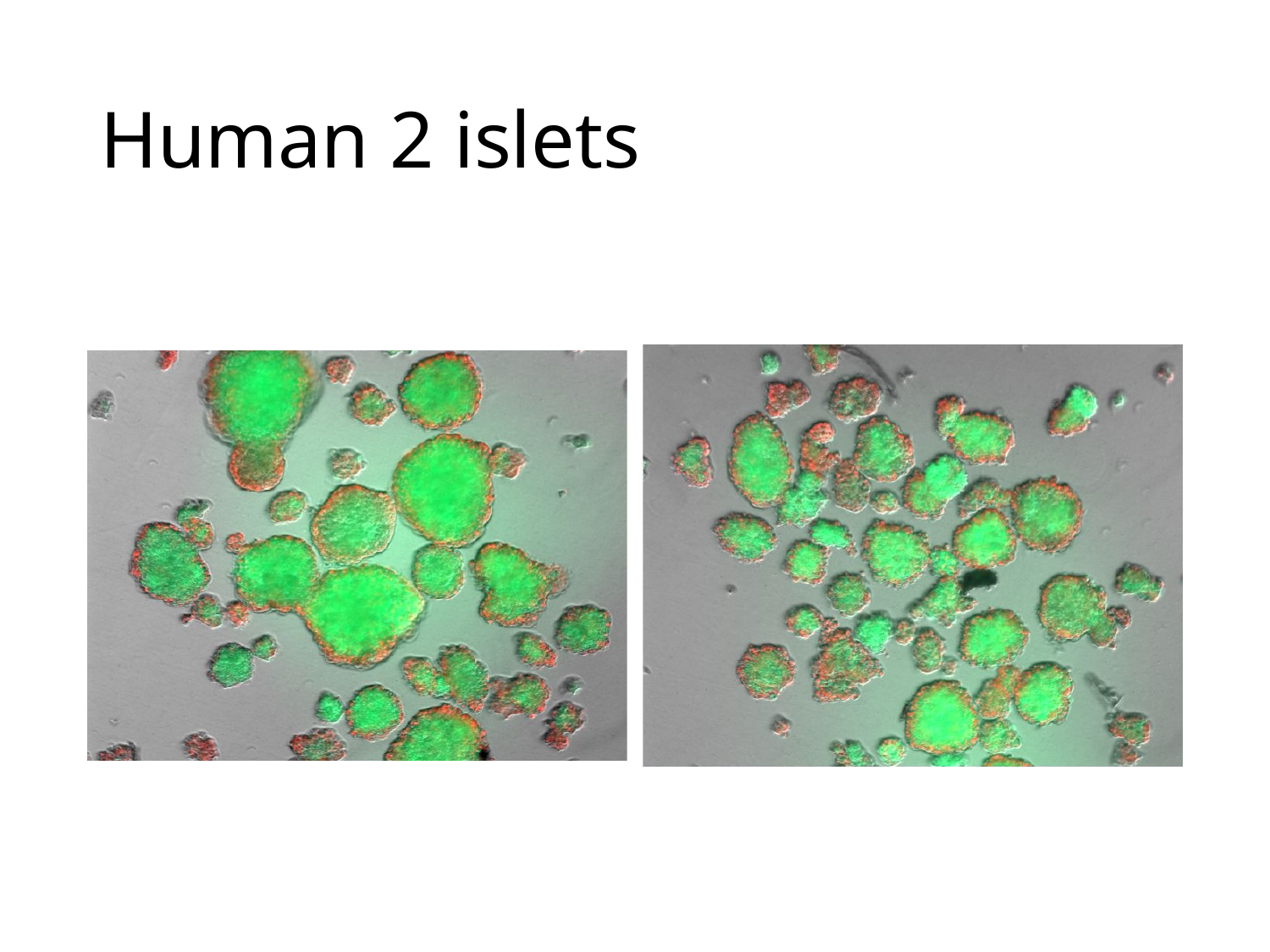

# Human 2 islets

## Slide 10
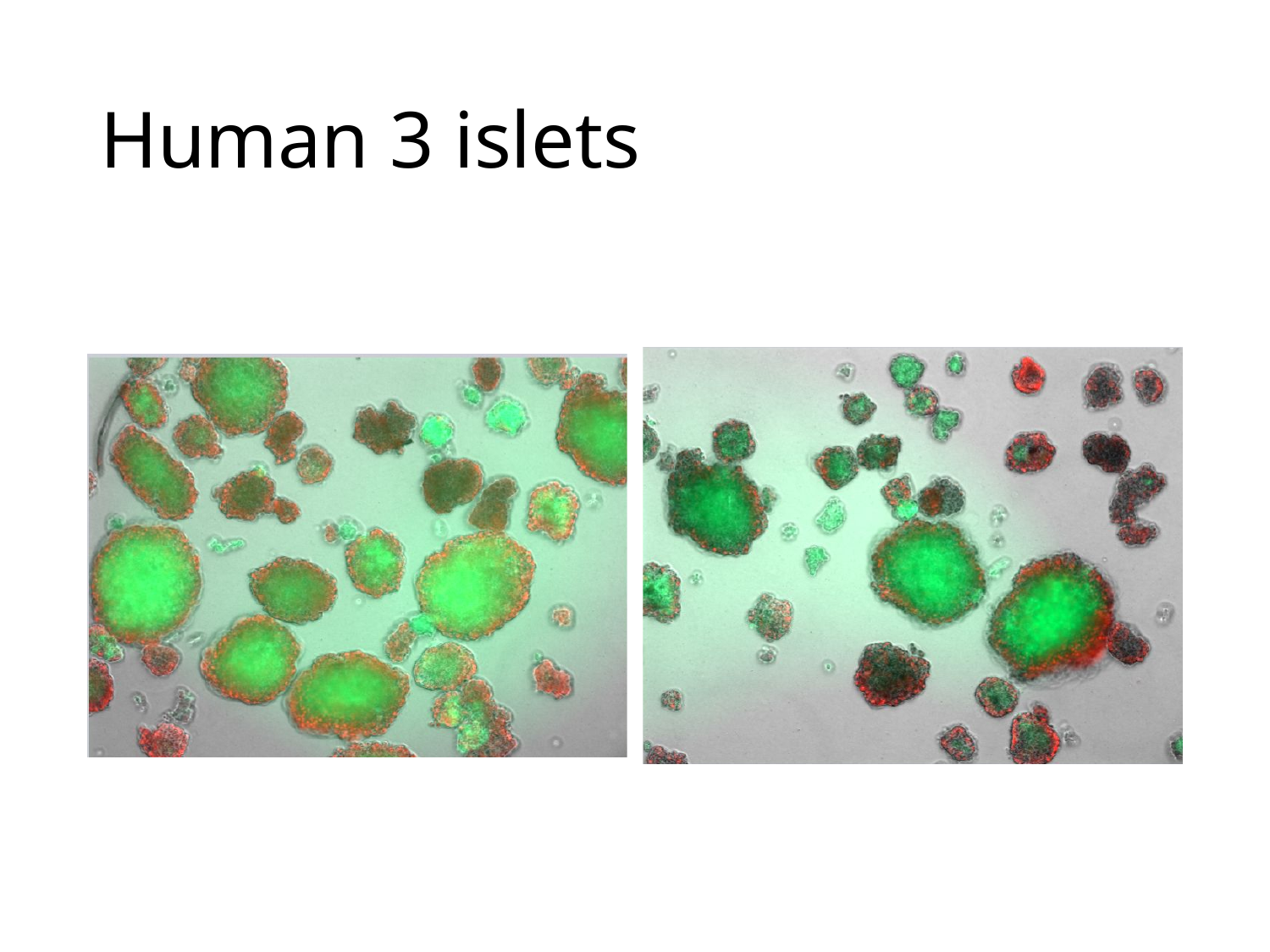

# Human 3 islets

## Slide 11
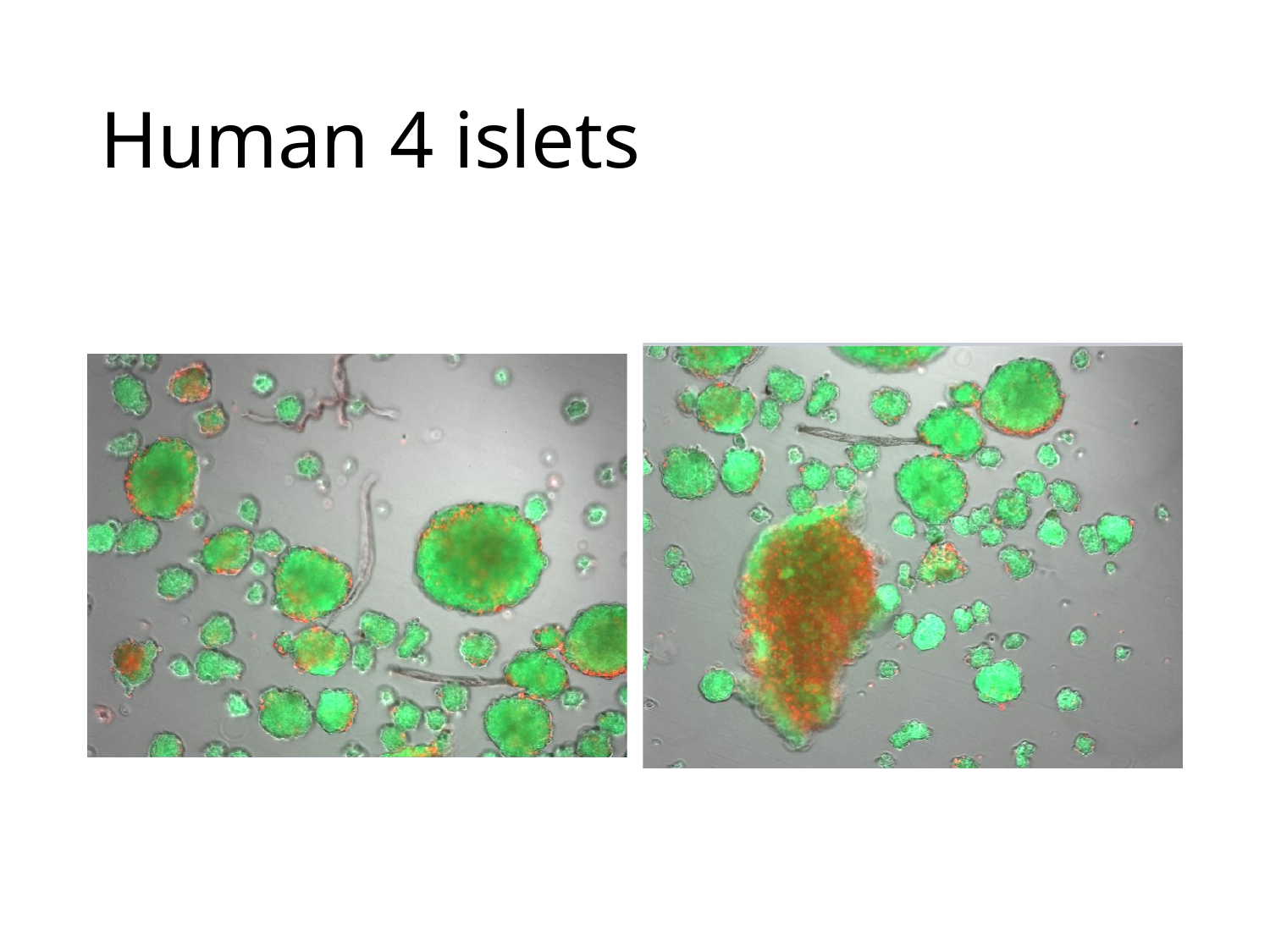

# Human 4 islets

## Slide 12
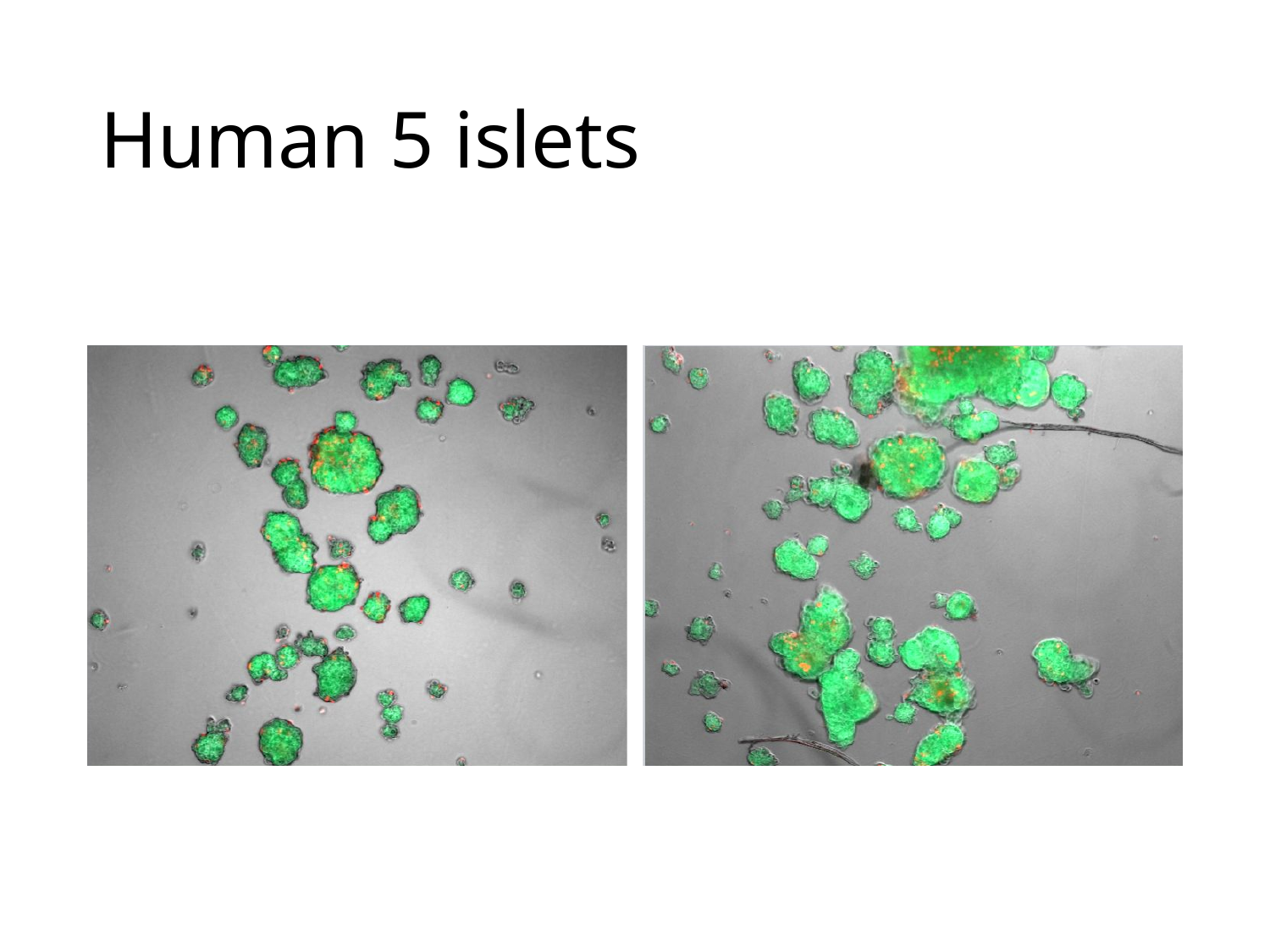

# Human 5 islets

## Slide 13
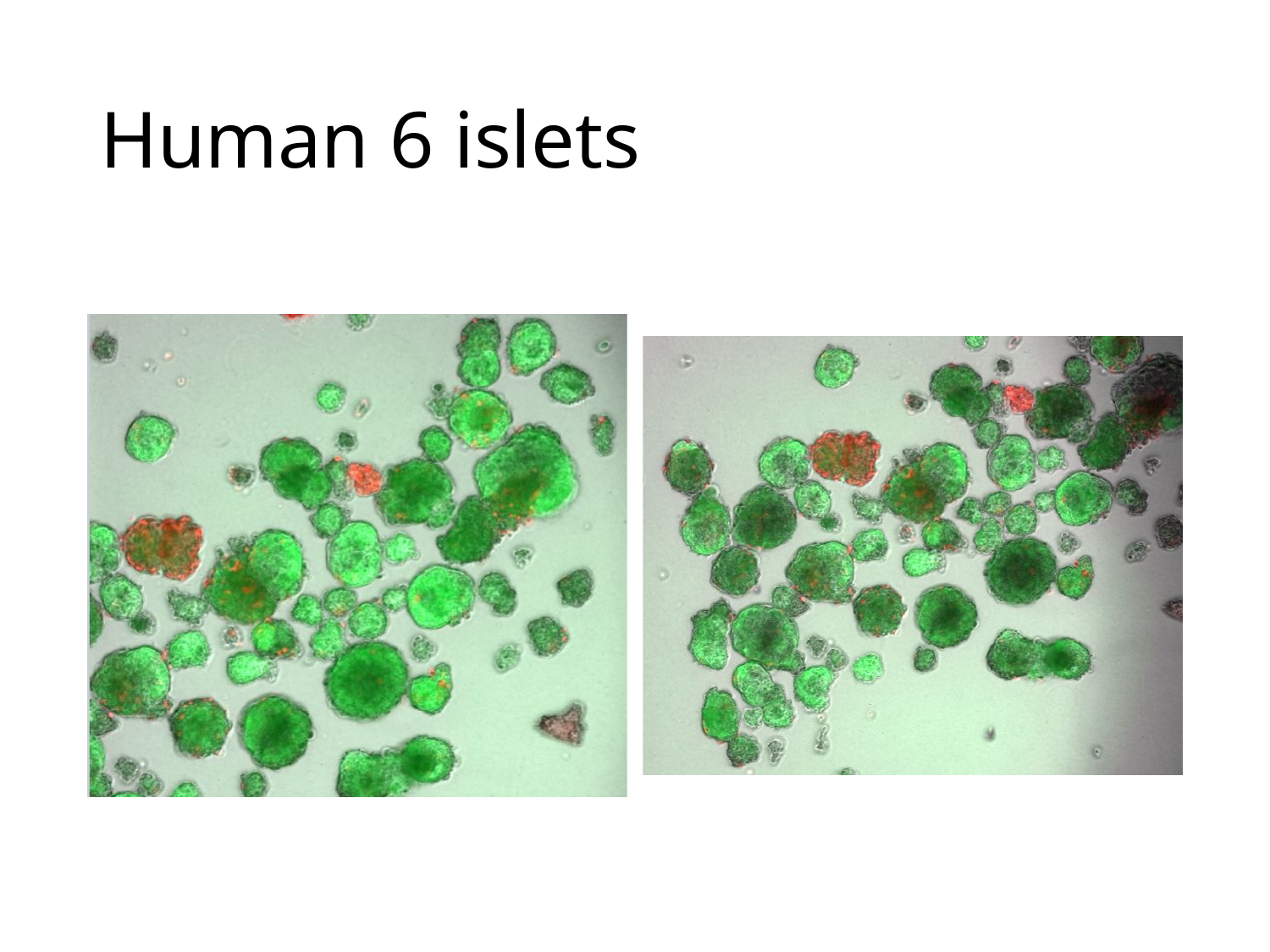

# Human 6 islets
