# Supplementary material for: Significant expansion of the donor pool achieved by utilizing islets of variable quality in the production of allogeneic “Neo-Islets”, 3-D organoids of Mesenchymal Stromal and islet cells, a novel immune-isolating biotherapy for Type I Diabetes
Source: PLoS One. 2023 Aug 24;18(8):e0290460. doi: 10.1371/journal.pone.0290460 (PMC10449143; doi:10.1371/journal.pone.0290460)
Supplement: S2 File — (PDF) [file pone.0290460.s004.pdf]

Checklist for Reporting Human Islet Preparations Used in Research

Adapted from Hart NJ, Powers AC (2018) Progress, challenges, and suggestions for using human islets to understand islet biology and human diabetes. Diabetologia https://doi.org/10.1007/s00125-018-4772-2

**Islet preparation**	**1**	**2**	**3**	**4**	**5**	**6**	**7**	**8[a]**
**MANDATORY INFORMATION**								
Unique identifier	HP-17346-01	HP-18047-01	HP-18054-01	HP-18066-01	HP-18095-01	HP-18200-01		
Donor age (years)	27	28	40	48	29	61		
Donor sex (M/F)	M	M	M	F	M	F		
Donor BMI (kg/m^2)	25.8	34.7	25.3	21.5	22.8	28.9		
Donor HbA_{1c} or other measure of blood glucose control	5.5	4.2	5.3	5.3	5.5	5.2		
Origin/source of islets[b]	Prodo Labs	Prodo Labs	Prodo Labs	Prodo Labs	Prodo Labs	Prodo Labs		
Islet isolation centre	Prodo Labs	Prodo Labs	Prodo Labs	Prodo Labs	Prodo Labs	Prodo Labs		
Donor history of diabetes? Please select yes/no from drop down list	No	No	No	No	No	No		
**If Yes, complete the next two lines if this information is available**								
Diabetes duration (years)								
Glucose-lowering therapy at time of death[c]								

Continues on the next page

RECOMMENDED INFORMATION								
Donor cause of death	Head Trauma	Head Trauma	Head Trauma	Stroke	Head Trauma	Head Trauma		
Warm ischaemia time (h)								
Cold ischaemia time (h)								
Estimated purity (%)	90	95	90-95	90	85	85		
Estimated viability (%)	80	80	70	90	95	90		
Total culture time (h)[d]								
Glucose-stimulated insulin secretion or other functional measurement[e]								
Handpicked to purity? Please select yes/no from drop down list								
Additional notes								

[a]If you have used more than eight islet preparations, please complete additional forms as necessary
[b]For example, IIDP, ECIT, Alberta IsletCore
[c]Please specify the therapy/therapies
[d]Time of islet culture at the isolation centre, during shipment and at the receiving laboratory
[e]Please specify the test and the results
